# Supplementary material for: Quantum dissipation driven by electron transfer within a single molecule investigated with atomic force microscopy
Source: Nat Commun. 2020 Mar 12;11:1337. doi: 10.1038/s41467-020-15054-w (PMC7067884; doi:10.1038/s41467-020-15054-w)
Supplement: Supplementary file 1 — Supplementary Information [file 41467_2020_15054_MOESM1_ESM.pdf]

Supplementary Information for

Quantum dissipation driven by electron transfer within a single molecule investigated with atomic force microscopy

Jan Berger[1]†, Martin Ondráček[1]†, Oleksandr Stetsovych[1]†, Pavel Malý[2], Petr Holý[3], Jiří Rybáček[3], Martin Švec[1,4], Irena G. Stará[3], Tomáš Mančal[2] , Ivo Starý[3], Pavel Jelínek[1,4]*

[1]*Institute of Physics of the Czech Academy of Sciences, Cukrovarnická 10, 16200 Prague 6, Czech Republic.*

[2]*Charles University, Faculty of Mathematics and Physics, Ke Karlovu 5, 121 16 Prague 2, Czech Republic*

[3]*Institute of Organic Chemistry and Biochemistry of the Czech Academy of Sciences, Flemingovo nám. 2, 16610 Prague 6, Czech Republic.*

[4]*Regional Centre of Advanced Technologies and Materials, Palacký University, Šlechtitelů 27, 78371 Olomouc, Czech Republic.*

*Correspondence to: jelinekp@fzu.cz

†All authors contributed equally to this work and can be considered as the first author.

Supplementary Note 1: Additional information about the formation of charge states and their characteristics

In this section, we provide additional information about the formation/annihilation of the single electron charging processes depending on an applied voltage and a tip position with respect to the molecule and limited possibility to discriminate the charge states in far tip-sample distances.

First, we describe an experiment, which demonstrates how the +1h charge state can be annihilated by a proper choice of tip position and applied bias voltage. Supplementary Figure 2 shows a sequence of constant height frequency shift and dissipation images acquired for different voltages. At bias voltage −2 V the molecule is in the +1h charge state, which is revealed by the presence of the characteristic line, see Supplementary Fig. 2a,d. In the next image, the bias voltage was reduced to a less negative value of −1.5 V while the tip height was kept constant. When the probe approaches the molecule, at one moment, an electron tunnels from the tip to the molecule and the hole is annihilated as shown on Supplementary Fig. 2b,e. Consequently, the sharp line disappears and the frequency shift becomes more negative, revealing large attractive force acting between tip and sample.

For the measurement shown on Supplementary Fig. 3, we first acquired a parabola in far distance (black line), where electron tunneling from tip to the molecule is not possible. Consequently, the charging process did not take place. After that, we approached the tip towards to the molecule by 1 nm and we swept the bias voltage from +4 to −4 V. In this case, we clearly observed two characteristic jumps in the Kelvin parabola revealing two charging events (see blue line). Next, we kept the bias voltage set to −4 V to maintain the molecule at +2h charge state and we retracted the tip to the initial tip sample distance by 1 nm. Finally, we acquired again the Kelvin parabola in this tip-sample distance (dashed red curve). We can see that the original and final Kelvin parabolas acquired in far distance are very similar and we cannot distinguish the presence of charge molecular states in such distances. We attributed this effect to the presence of strongly polar NaCl substrate, which may efficiently screen the local point charges placed on the surface. Unfortunately, signal-to-noise ratio of our instrument does not allow to detect variation of the frequency shift in orders of 0.01 Hz, which would reveal the +1h charge state in such far distances. This makes distinguishing the charge state of the molecule very difficult in far distances.

The lack of resolution in the far distance is actually already obvious from the vertical cut of frequency shift displayed on Fig. 2g of the main text. It is evident that the frequency shift signal fades away quickly when increasing the tip-sample distance. Thus characteristic round features indicating the position of the two ferrocene units are only visible in close distances, as one can see from Fig. 2g (*x*-*y* constant height and vertical *x*-*z* Δf images, where you can see bisFc molecule and singularity line). In tip-sample distances above the dissipation line, the frequency shift signal is below the noise-level and we cannot make any statement about the charge state of the molecule.

In far distance, we are no more sensitive to the presence of charge states, as discussed previously. The variation of the frequency shift contrast at different tip heights can be seen from Supplementary Fig. 4. First we scan the molecule in neutral state (U = −0.1 V) in close distance, after that we switch the molecule to +1h charge state (U = −2 V) but keeping the tip height constant. The presence of the +1h state is clearly visible by the presence of the sharp line in both channels. After that, we retracted the tip 0.8 nm away and we lowered the bias voltage to 0.1 V,

which in close distances would quench immediately the molecule to the neutral state. Nevertheless, the large tip-sample distance prevents the electron tunneling from the tip to the molecule and therefore the molecule remains in the +1h charge state. This is confirmed by the presence of the characteristic sharp line. However, we no more see the circular features representing ferrocene units in the frequency shift and the signal becomes monotonous except for the sharp line. Finally, we decreased the tip-sample distance and the molecule becomes neutral again. The fact that we can also modulate the position of the charge within the molecule—as revealed by the presence of the sharp line—even at low bias demonstrates that the electrostatic interaction is mainly driven by the presence of a static charge on the tip, but not by an induced one.

Supplementary Note 2: Description of the numerical model

The oscillating tip of the atomic force microscope indirectly measures forces between the tip and the sample through the shift Δf of resonant frequency f_r at which the cantilever with the tip oscillates. In addition, energy dissipated in the course of the oscillation can be detected and used to characterize (the non-conservative component of) the forces. Assuming a stiff cantilever, so that the tip motion, represented by time varying tip height z_t, is only very weakly perturbed by the external forces and can be well approximated by

$$z_{\mathrm{t}}(t) = z_0 + A\cos(2\pi f_{\mathrm{r}} t)\,, \tag{1}$$

the frequency shift Δf is related to the force F as

$$\Delta f = -\frac{f_{\mathrm{r}}^2}{kA}\int_0^{\frac{1}{f_{\mathrm{r}}}} F(t)\cos(2\pi f_{\mathrm{r}} t)dt \tag{2}$$

and the dissipated energy E_{diss} is

$$E_{\mathrm{diss}} = 2\pi f_{\mathrm{r}} A\int_0^{\frac{1}{f_{\mathrm{r}}}} F(t)\sin(2\pi f_{\mathrm{r}} t)\,dt. \tag{3}$$

In the above, A denotes the oscillation amplitude, f_r the resonant frequency and $F(t)$ the time-dependent force. It is only a matter of convention that we chose the cosine function to describe the tip oscillation, rather than e.g. the sine function or a sine function with some general phase-shift. This choice just fixes the origin of the time scale, $t = 0$. In the following, we will only be interested in the components of $F(t)$ which are related to the extra charge localized on the molecule. All other components of the force are typically conservative, hence not contributing to E_{diss}, and only contribute a smooth featureless background to Δf. The charge-dependent component of the force F_{Q} is

$$F_{\mathrm{Q}}(t) = p_{\mathrm{A}}(t)F_{\mathrm{A}}\left(x_{\mathrm{t}}, y_{\mathrm{t}}, z_{\mathrm{t}}(t)\right) + p_{\mathrm{B}}(t)F_{\mathrm{B}}\left(x_{\mathrm{t}}, y_{\mathrm{t}}, z_{\mathrm{t}}(t)\right), \tag{4}$$

where F_A (F_B) denote the electrostatic force felt by the tip because of the extra charge +1h at location A (B) in the molecule; p_A and p_B are the time-dependent probabilities that the respective locations A, B are occupied by the charge,

$$p_{\mathrm{A}} + p_{\mathrm{B}} = 1\,, \tag{5}$$

and the coordinates x_t, y_t, z_t specify the position of the tip.

If we represent the electrostatic effects of the tip by a single point charge Q_t placed at (x_t,y_t,z_t), the forces F_A and F_B are given by

$$F_{\mathrm{A}} = \frac{eQ_{\mathrm{t}}(z_{\mathrm{t}} - z_{\mathrm{A}})}{4\pi\epsilon_0[(x_t - x_{\mathrm{A}})^2 + (y_{\mathrm{t}} - y_{\mathrm{A}})^2 + (z_{\mathrm{t}} - z_{\mathrm{A}})^2]^{3/2}}\,, \tag{6a}$$

$$F_{\mathrm{B}} = \frac{eQ_{\mathrm{t}}(z_{\mathrm{t}} - z_{\mathrm{B}})}{4\pi\epsilon_0[(x_{\mathrm{t}} - x_{\mathrm{B}})^2 + (y_{\mathrm{t}} - y_{\mathrm{B}})^2 + (z_{\mathrm{t}} - z_{\mathrm{B}})^2]^{3/2}}\,. \tag{6b}$$

Note that only the z component of the Coulombic force is relevant for the motion of the tip. The electrostatic interaction of such tip then shifts the energies that correspond to the charge localized at sites A or B. These energy shifts are again dictated by the Coulomb interaction, so

$$E_{\mathrm{A}} = \frac{eQ_{\mathrm{t}}}{4\pi\epsilon_0\sqrt{(x_{\mathrm{t}} - x_{\mathrm{A}})^2 + (y_{\mathrm{t}} - y_{\mathrm{A}})^2 + (z_{\mathrm{t}} - z_{\mathrm{A}})^2}}\,, \tag{7a}$$

$$E_{\mathrm{B}} = \frac{eQ_{\mathrm{t}}}{4\pi\epsilon_0\sqrt{(x_{\mathrm{t}} - x_{\mathrm{B}})^2 + (y_{\mathrm{t}} - y_{\mathrm{B}})^2 + (z_{\mathrm{t}} - z_{\mathrm{B}})^2}}\,, \tag{7b}$$

respectively.

When the charge on the molecule switches from one location to another, the molecule reacts by reorganizing its structure. The reorganization in general involves both a shift in positions of atomic nuclei as well as a redistribution of electron clouds which takes place in order to partially screen the localized charge. Let us assume that the slowest component of the reorganization occurs at a time scale comparable to the tip oscillation and that it can be characterized by a single reaction coordinate, which we are going to denote q; see Fig. 3a,b in the main text. The energy landscape in q will be assumed to be described by a parabolic potential $V_A(q)$ or $V_B(q)$, the minimum of which depends on whether the molecular charge resides at site A or B:

$$V_{\mathrm{A}} = \frac{1}{2}m_q\omega_q^2(q + \delta/2\,)^2\,, \tag{8a}$$

$$V_{\mathrm{B}} = \frac{1}{2} m_q \omega_q^2 (q - \delta/2\,)^2\,, \tag{8b}$$

where δ is the distance in the q between the equilibria that correspond to sites A and B, respectively, being occupied; m_q is the effective mass and ω_q the angular frequency of the oscillator formed by the potential wells $V_{\mathrm{AB}}(q)$. We used the freedom to arbitrarily define the origin of the q axis in order to place the potentials V_{A} and V_{B} in a symmetric way with respect to the origin. We are going to characterize the statistical state of the molecule by probability densities $\rho_{\mathrm{A}}(q,t)$ and $\rho_{\mathrm{B}}(q,t)$,

$$p_{\mathrm{A}}(t) = \int \rho_{\mathrm{A}}(q,t)dq\,, \tag{9a}$$

$$p_{\mathrm{B}}(t) = \int \rho_{\mathrm{B}}(q,t)dq\,, \tag{9b}$$

The q-dependent energy change accompanying the switch of the charge location from B to A is

$$\Delta E_{\mathrm{AB}}(q) = E_{\mathrm{A}}(x_{\mathrm{t}}, y_{\mathrm{t}}, z_{\mathrm{t}}) - E_{\mathrm{B}}(x_{\mathrm{t}}, y_t, z_{\mathrm{t}}) + V_{\mathrm{A}}(q) - V_{\mathrm{B}}(q) + \Delta E_0, \tag{10}$$

where ΔE_0 is the initial energy difference between the two possible charge positions A and B (with the molecular structure in the corresponding relaxed configuration for both of these charge positions) in the absence of the tip. A non-zero initial energy difference ΔE_0 is a consequence of a slight asymmetry of the molecule adsorbed on the insulating surface. Such an asymmetry is necessarily present because the distance of the Fe redox centers in the molecule is incommensurate with respect to the NaCl(100) surface structure, so the two parts of the molecule with the respective redox centers will adsorb in a slightly different geometry and they will experience different electrostatic fields and dielectric screening from the ionic substrate. The immediate energy difference $\Delta E_{\mathrm{AB}}(q,t)$ is time dependent through its dependence on tip position, in particular the vertical tip coordinate $z_{\mathrm{t}}(t)$. Note that the quantity $\Delta E(t)$, which appears in equation (2) of the main paper, collects those terms of $\Delta E_{\mathrm{AB}}(q,t)$ that depend on the position of the redox centers with respect to both the tip and the substrate: $\Delta E(t) = E_{\mathrm{A}} - E_{\mathrm{B}} + \Delta E_0$.

We model the dynamics of the probability densities in the potential wells by the Smoluchowski diffusion equation in order to mimic the relaxation of the molecule towards its equilibrium configuration for a given charge location. Additionally, q-preserving charge hopping from A to B and back will be allowed with rates k_{BA} and k_{AB} [as in Valkunas, L., Chmeliov, J., Krüger, T. P. J., Ilioaia, C. & Grondelle, R. *JPC Lett.* **3**, 2779–2784 (2012)]. The resulting coupled differential equations for $\rho_{\mathrm{A}}(q,t)$ and $\rho_{\mathrm{B}}(q,t)$ (adapted from Mukamel: *Principles of Nonlinear Optical Spectroscopy* book, Eq. 12.48) become

$$\frac{\partial \rho_{\mathrm{A}}}{\partial t} = \frac{k_{\mathrm{B}}T}{m_q \gamma} \frac{\partial^2 \rho_{\mathrm{A}}}{\partial q^2} + \frac{m_q \omega_q^2}{\gamma} \frac{\partial}{\partial q}\left(q + \frac{\delta}{2}\right) \rho_{\mathrm{A}} + k_{\mathrm{AB}}(q,t)\rho_{\mathrm{B}} - k_{\mathrm{BA}}(q,t)\rho_{\mathrm{A}}\,, \tag{11a}$$

$$\frac{\partial \rho_{\mathrm{B}}}{\partial t} = \frac{k_{\mathrm{B}}T}{m_q \gamma} \frac{\partial^2 \rho_{\mathrm{B}}}{\partial q^2} + \frac{m_q \omega_q^2}{\gamma} \frac{\partial}{\partial q}\left(q - \frac{\delta}{2}\right) \rho_{\mathrm{B}} - k_{\mathrm{AB}}(q,t)\rho_{\mathrm{B}} + k_{\mathrm{BA}}(q,t)\rho_{\mathrm{A}}\,, \tag{11b}$$

where k_B is the Boltzmann constant, T is the thermodynamic temperature of the substrate with which the molecule equilibrates, γ is the friction constant for the reorganization coordinate, and the hopping rates are

$$k_{AB}(q,t) = k_0 \exp\left\{-\frac{[E_{AB}(q,t) + \Lambda]^2}{4\Lambda k_B T}\right\}. \tag{12a}$$

$$k_{BA}(q,t) = k_0 \exp\left\{-\frac{[E_{AB}(q,t) - \Lambda]^2}{4\Lambda k_B T}\right\}. \tag{12b}$$

Supplementary Equation (12b) corresponds to equation (2) from the main paper, while (12a) gives the hopping rate in the opposite direction. The parameter Λ in the above equations is the part of reorganization energy that pertains to degrees of freedom (DOF) which relax much faster than those represented by coordinate q. These fast DOF are those which we dubbed "Marcus" DOF, while those represented by q are the "explicit" DOF. The coefficient k_0 scales with temperature and Λ according to

$$k_0 = \frac{|M|^2}{\hbar}\sqrt{\frac{\pi}{\Lambda k_B T}}\ . \tag{13}$$

The notation in Supplementary Eqs. (11) can be simplified if we introduce a characteristic time constant K_Λ for the diffusion and a temperature-dependent diffusion parameter $D(T)$ by

$$K_\Lambda = \frac{\omega_q^2}{\gamma} \tag{14}$$

and

$$D(T) = \frac{k_B T}{m_q \omega_q^2}\ . \tag{15}$$

The coupled dynamical equations for the probability densities then become

$$\frac{\partial \rho_A}{\partial t} = K_\Lambda\left[D(T)\frac{\partial^2}{\partial q^2} + \frac{\partial}{\partial q}\left(q + \frac{\delta}{2}\right)\right]\rho_A + k_{AB}(q,t)\rho_B - k_{BA}(q,t)\rho_A\ , \tag{16a}$$

$$\frac{\partial \rho_B}{\partial t} = K_\Lambda\left[D(T)\frac{\partial^2}{\partial q^2} + \frac{\partial}{\partial q}\left(q - \frac{\delta}{2}\right)\right]\rho_B - k_{AB}(q,t)\rho_B + k_{BA}(q,t)\rho_A\ . \tag{16b}$$

The number of independent parameters in the above equations can be further reduced by rescaling q according to $\tilde{q} = q/\delta$ and introducing a new parameter

$$E_\Lambda = \frac{m_q \omega_q^2 \delta^2}{2}\ . \tag{17}$$

The probability densities shall be rescaled accordingly, $\tilde{\rho}_A(\tilde{q},t) = \rho_A(q,t)\delta$ and $\tilde{\rho}_B(\tilde{q},t) = \rho_B(q,t)\delta$. Supplementary Eqs. (16) can now be rewritten as

$$\frac{\partial \rho_A}{\partial t} = K_\Lambda \left[\frac{k_B T}{2E_\Lambda}\frac{\partial^2}{\partial q^2} + \frac{\partial}{\partial q}\left(q + \frac{1}{2}\right)\right]\rho_A + k_{AB}(q,t)\rho_B - k_{BA}(q,t)\rho_A\,, \qquad (18a)$$

$$\frac{\partial \rho_A}{\partial t} = K_\Lambda \left[\frac{k_B T}{2E_\Lambda}\frac{\partial^2}{\partial q^2} + \frac{\partial}{\partial q}\left(q - \frac{1}{2}\right)\right]\rho_B - k_{AB}(q,t)\rho_B + k_{BA}(q,t)\rho_A \qquad (18b)$$

and the expressions for the potential wells, Supplementary Eqs. (8), as

$$V_A = E_\Lambda\left(q + \tfrac{1}{2}\right)^2, \qquad (19a)$$

$$V_B = E_\Lambda\left(q - \tfrac{1}{2}\right)^2. \qquad (19b)$$

Although we have omitted the tildes in the above Supplementary Eqs. (18) and (19), it was already written in the rescaled quantities. We may interpret the new parameter E_Λ as a reorganization energy associated with the relatively slow dynamics in coordinate q (in contrast to the reorganization energy Λ, associated with the "faster" reaction coordinates). The dynamics of the electron transfer (ET) is then essentially characterized by four parameters (apart from the temperature, which we assume to be known from measurement): the diffusion time scale K_Λ, the slow reorganization energy E_Λ, the fast reorganization energy Λ, and the hopping-rate coefficient k_0 (or, equivalently, the transfer matrix element M).

Supplementary Note 3: Sensitivity of the line shape of $\Delta f(z)$, $E_{diss}(z)$ signals to the electrostatic model of the tip

The shape and spatial location of the sharp linear feature related to the electron transfer is dictated by the interaction of the charged centers in the molecule with the tip of the microscopic sensor and with the substrate. Electrostatic properties of the tip termination are the decisive factor here. Supplementary Figure 5 demonstrates the dependence of the line shape on the tip by showing how the shape of the imaged feature changes after the tip is intentionally modified by bringing it to a close contact with the substrate and the same molecule is then rescanned with the modified tip. Supplementary Figure 6 shows the same effect for the simulated data. Asymmetric tips are modelled with two-point charges on the apex instead of just one.

Supplementary Note 4: Numerical details regarding simulation of ET dynamics

According to Supplementary Eqs. (12), ET is most probable to occur when $\Delta E_{AB}(q, t) = \pm\Lambda$ (the sign depending on the direction of the transfer). In such a situation, the energy dissipated instantaneously (into the "Marcus" DOF) as the charge hops from one site to another equals the

reorganization energy Λ. Another contribution to the dissipation of energy E_{diss} is the damped relaxation to the ground state in the parabolic potentials (the explicit DOF). This contribution scales up with the other reorganization energy E_Λ and also increases with the diffusion time constant K_Λ as the relaxation gets faster with larger K_Λ.

We have carefully chosen the parameters of the numerical model in order to reproduce the experimental conditions and the observed signal as faithfully as possible. The resonance frequency and stiffness of the sensor, $f_r = 1$ MHz, $k = 1$ MNm^{-1}, were dictated by the known properties of the measuring device. All simulations presented here were done for an amplitude of $A = 40$ pm, for which we achieved the best sensitivity in the experiment (an optimal trade-off between a signal to noise ratio, which improves with increasing amplitude, and a loss of spatial sensitivity for larger amplitudes). The electric charge on the tip apex, $Q_t = +0.25\ e$ (where e is the elementary charge) was chosen in order to roughly reproduce the typical magnitude of the measured signal on the sharp features related to charge hopping (frequency shift in the order of 0.1 Hz, dissipated energy several meV). The initial (tip-independent) site asymmetry of $\Delta E_0 = 10^{-20}$ J ≈ 62.4 meV then nicely reproduces the curved shape of the linear feature observed in some of the experiments; see Fig. 2g,h, cf. simulation on Fig. 4a,b.

Given the choices made so far, the parameters which directly relate to the dynamics of the electron transfer were adjusted with the following conditions in mind: (i) The effective ET rate $\langle k_{ET} \rangle$ derived from the experimentally-determined ratio of the frequency shift signal to the dissipation signal according to equation (1) of the main paper fell, as Fig. 2k testifies, into the range from 2×10^6 s^{-1} up to about 10^7 s^{-1}, with the exception of one data set that went up to about 1.8×10^7 s^{-1}. We tried to get the same order of magnitude for the corresponding quantity derived from the simulated signal. (ii) The results should be rather insensitive to temperature; refer again to Fig. 2k to see this is the case for the measured values when changing the temperature from about 5 K to about 1 K. (iii) The shape of peak profiles in z-scans as seen in both signal channels, frequency shift as well as dissipation, should be a bell-like curve resembling a Gaussian or Lorentzian function (these two are not distinguishable within experimental precision) rather than being almost rectangular or semi-circle shaped, as it could happen with an inappropriate parameter choice in our model. Although we were not able to make a uniquely optimal choice of the parameters, here we give the values that fulfilled the above criteria reasonably well. The reorganization energy for the explicit degrees of freedom $E_\Lambda = 4\times10^{-23}$ J ≈ 2.5 meV, relaxation rate of the explicit DOF $K_\Lambda = 2\times10^6$ s^{-1}, Marcus reorganization energy $\Lambda = 4\times10^{-22}$ J ≈ 2.5 meV, the transfer rate $k_0 = 7.07\times10^7$ s^{-1} for the temperature of $T = 1.2$ K and $k_0 = 3.46\times10^7$ s^{-1} for the temperature of $T = 5$ K, which corresponds to the transfer matrix element $M = 3.65$ μeV. These values are adopted to obtain the results shown in Figures 3 and 4 in the main text.

The above choice of parameters K_Λ and E_Λ for the ET dynamics corresponds to a reorganization intermediate rate of the explicit DOF relaxation. In this case, the relaxation of the explicit DOF takes place on a time scale comparable to the oscillation period of the AFM tip. Let us now compare our full model with the above parameters (at 5 K) to a simplified version that is based solely on the Marcus model for charge hopping while it completely disregards the explicit degree of freedom. Within such model, the probabilities that the charge is located at a particular position evolve according to

$$\frac{\partial p_A}{\partial t} = p_B k_0 \exp\left\{\frac{-[\Delta E(t) + \Lambda]^2}{4\Lambda k_B T}\right\} - p_A k_0 \exp\left\{\frac{-[\Delta E(t) - \Lambda]^2}{4\Lambda k_B T}\right\}, \qquad (20a)$$

$$\frac{\partial p_B}{\partial t} = p_A k_0 \exp\left\{\frac{-[\Delta E(t) - \Lambda]^2}{4\Lambda k_B T}\right\} - p_B k_0 \exp\left\{\frac{-[\Delta E(t) + \Lambda]^2}{4\Lambda k_B T}\right\}. \qquad (20b)$$

For either very fast ($K_\Lambda >> f_r$) or slow ($K_\Lambda << f_r$) explicit DOF relaxation, the Marcus model would be able to reproduce the results of the full model almost perfectly in both signal channels, the frequency shift as well as dissipated energy E_{diss}. Even in the intermediate case, we can still roughly reproduce the model with the explicit DOF by the Marcus model if we pick an appropriate value of reorganization energy Λ to be inserted into Supplementary Eq. (20), but the shape of the Δf, E_{diss} peaks is more rounded. In Supplementary Figure 11a,b, we compare the results from the full model to the simplified all-Marcus model. The reorganization energy for the all-Marcus model was chosen to be $\Lambda = 6\times10^{-22}$ J ≈ 3.75 meV and the transfer rate was set to $k_0 = 2.45\times10^7$ s^{-1} (at $T = 5$ K). Note that the value of Λ was chosen to lie in between Λ and $\Lambda + E_\Lambda$ of the full model. Such a choice ensures an approximate agreement of the dissipated energy E_{diss} between the two models: The typical dissipation energy E_{diss} as a result of one charge-transfer event in the Marcus model is roughly the reorganization energy Λ. In the full model, on the other hand, the typical dissipated energy E_{diss} equals the complete reorganization energy of the fast (Marcus) DOF (Λ) plus part of the reorganization energy of the slower explicit DOF (E_Λ). The reason why only part of the reorganization energy of the explicit DOF dissipates is that these slower DOF do not have the time to fully reorganize before next electron transfer occurs. In spite of a crude agreement of signal magnitudes, peak shapes E_{diss} clearly differ between the full and the simplified Marcus model. The full model with the explicit DOF reproduces the shape of the E_{diss} peaks in experimentally detected signal somewhat better.

Yet more prominent discrepancies in peak shapes arise with respect to the experiment if we used another, extremely simplified ET model. This model consists in the assumption that charge the immediately switches from one site A to B as soon as a difference in energies corresponding to these sites, $\Delta E(t) = E_A - E_B + \Delta E_0$, surpasses a certain constant threshold δE. In the same way, the charge moves from B to A as soon as $\Delta E(t) < -\delta E$. When $-\delta E < \Delta E(t) < +\delta E$, no electron transfer can happen. Simulated $\Delta f(z)$, $E_{diss}(z)$ profiles and their comparison to the full model are shown in Supplementary Fig. 11c,d; choosing $\delta E = 4\times10^{-22}$ J ≈ 2.5 meV as the threshold energy. In this *threshold model*, the dissipated energy E_{diss} in one cycle is always either $2\delta E$ or zero. Thus, the dissipation signal E_{diss} appears only when the oscillating tip modulates the energy difference $\Delta E(t)$ between the two redox sites larger than the threshold energy δE during tip approach and retraction and consequently the electron transfer can proceed in both directions. On the contrary, the zero-dissipation case occurs if the tip-induced energy variation $\Delta E(t)$ does not suffice to drive the electron transfer. This on/off character results in rectangular shapes of the peaks in the dissipation E_{diss} channel of the measured signal, as shown on Supplementary Fig. 11d. So on the one hand, by comparing simulated $\Delta f(z)$ profiles for three different ET models, see Supplementary Figs. 11a,c, we can realize that the shape of $\Delta f(z)$ is rather insensitive to the ET models. On the other hand, it is evident that the shape of E_{diss} peaks is sensitive to the ET dynamics. Thus, it can provide more insight into the ET dynamics. In order to relate the shape of the $E_{diss}(z)$ peaks to ET rates, we plot in Supplementary Fig. 12 the mean number of ET events that take place during one oscillation cycle of AFM probe for each model. Variability of transfer probability peak

shapes in these plots correlates very well with the variations of the dissipated energy $E_{diss}(z)$ profiles. This demonstrates the direct relation between the ET dynamics and the shape of $E_{diss}(z)$ profiles.

Supplementary Note 5: Additional discussion on the explicit and Marcus DOF

A better agreement of our full numerical model with the experimental evidence as compare to what we call the Marcus model, reveals the importance of the explicit DOF to describe the electron transfer process. The better agreement concerns the calculated line shapes and their amplitude dependence. On the other hand, the very existence of energy dissipation and its weak temperature dependence could have been explained by the Marcus model as well.

In principle, there are two components which define the out-of-equilibrium state of our system. They can be identified in the time evolution of the wave packets shown in Figure 3 in the manuscript.

The first component is related to a non-zero occupation probability p_A or p_B of a site that is higher in energy. This component is also included in the Marcus model and it is related to dynamical motion of the probe and to a finite value of the transfer rates k_{AB} and $k_{BA.}$ Here, in principle, the time scale of the DOF, into which the energy dissipates simultaneously with the charge transfer, is faster than the probe oscillation. The dissipation mechanism is related to irreversible energy transfer from the molecule to the substrate. We expect that this fast part comprises mostly of the intramolecular vibrational modes of the studied molecule, the near equilibrium motion (phonons) of the substrate, etc.

The second component, which defines the out-of-equilibrium condition, consists of a location of the center of mass of the wave packets out of the minimum of the parabolas. This component is only present in the full model. Importantly, this explicit DOF additionally modifies the occupation probability. Consequently, it also influences the line shape of the frequency shift and the dissipation signal, which allows to improve the agreement with the experiment. The comparison of the numerical model to experiment suggests the presence of some slow component of the bath dynamics. We do not have any definite physical explanation regarding its origin. It could be related, for instance, to the change of adsorption site of the molecule on NaCl or to a complex structural reorganization of the ionic substrate. Actually, the latter possibility is supported by the DFT calculations of charged molecules, showing pronounced relaxations of the substrate (bond length variation larger than 0.1 Å). In general, there may be other degrees of freedom than just the electron location that are driven out of equilibrium by the tip-oscillation driven charge transfer.

Supplementary Note 6: Other possible numerical models of the system

Finally, we would like to note that the numerical model we presented in this work may not be the unique way how to describe the weakly dependent ET process in our system. For example, the transfer rate as derived by J. Jortner [*J. Chem. Phys.* **64**, 4860–4867 (1976)] is temperature independent in the low temperature limit. However, in this model all modes are effectively frozen

and there is no dissipation. We have considered Jortner theory in an early stage of our theoretical analysis of the experiment, but the lack of dissipation excludes it from being the right answer to our particular problem. In our particular case, the explanation does not require temperature independent rates, rather the discrepancy with respect to equilibrium predictions arises from non-equilibrium (driven) state of our system. It is important to note, that if we assume that all the components of the environment relax fast on the time scale of the electron transfer process, i.e. we can apply Marcus theory to all degrees of freedom of the environment, the calculation still gives reasonable results. We can see however, that a more general theory, which allows for some degrees of freedom of the environment to relax relatively slowly, gives better agreement with the measured and amplitude dependence.

In principle, other formalisms designed to describe the dynamics of the open quantum systems such as Caldeira-Leggett could be applied to the description of the experiment. We hope the presented experimental data may stimulate further research activities applying methods describing quantum open systems to this system.

Supplementary Note 7: Background subtraction of $\Delta f(z)$ in the numerical model

In order to compare experimental and simulated data, it is essential to subtract the background on which the frequency dips are superimposed in the $\Delta f(z)$ spectra, because the sources of the background differ between the experiment and the simulations. We have described how we processed the experimental spectra in the Methods section of our paper. Here, we discuss the issue of the background subtraction for the simulated $\Delta f(z)$ spectra. Our numerical model does not contain any long-range van der Waals forces, so we do not need to subtract them. However, there is still a force originating from the point charge sitting on the molecule. Only the periodically tip-driven hopping of this charge between two redox centers causes the peaks, while the frequency shift related to the *z*-gradient of a static force that originates from the charge sitting on one particular site should be considered part of the background.

In the next, we describe a procedure to get rid of this background component of the simulated frequency shift. From the simulation, we determined time averages of occupation probabilities $p_A(t)$ and $p_B(t)$. This was done separately for each point of the spectrum, that means for each value of height z_0 around which the tip oscillated. Afterwards, we modelled the frequency shift again, imposing a constant fractional charges on site A and B, which correspond to the time-averaged values of p_A and p_B, respectively. We took this frequency shift $\Delta f(z)$ profile to be our background and we subtracted it from the frequency shift $\Delta f(z)$ given by our original simulation. The reader can convince oneself in Fig. 3c,d or Supplementary Fig. 9 that the simulated spectra consist exclusively of the peaks surrounded by pure zero signal, thus comparing well to the background-subtracted curves from the experiment.

Supplementary Note 8: Relation between the effective transfer rate $\langle k_{ET} \rangle$ and time dependent rates k_{AB}, k_{BA}

Our definition of the effective transfer rate $<k_{ET}>$, Eq. (1), follows analogous expressions that were independently suggested by the group of P. Grutter [Roy-Gobeil, A., Miyahara, Y. & Grutter, P. *Nano Lett.* **15,** 2324–2328 (2015)], their Eq. (6), and ourselves [Ondráček, M., Hapala, P. & Jelínek, P. *Nanotechnology* **27,** 274005 (2016)]. The applicability of this equation is limited to cases with a pronounced dissipation. This condition is only fulfilled when the switching rate is similar to the oscillation frequency f_o of the probe. In other words, the dissipation signal becomes maximized when switching occurs only a few times during one oscillation cycle and when the time-dependence of the occupancy of two possible states is in phase delay with respect to the oscillation amplitude.

The effective rate $<k_{ET}>$ relates to the phase shift between the tip oscillation and the electrostatic force $F_Q(t)$ sensed by the probe. Tip oscillation directly translates into periodic variations of the energy level difference $\Delta E(t) = E_A(x_t, y_t, z_t) - E_B(x_t, y_t, z_t)$, while $F_Q(t)$ is given by the immediate location of the hopping charge. Continuous variation of the energy level difference $\Delta E(t)$ results into temporal variation of the charge occupancies on sites A and B, i.e. $p_A(t)$, $p_B(t)$, but with a certain time delay with respect to the oscillation amplitude of the probe due to finite charge transfer rates k_{AB} and k_{BA}. This delay causes a phase shift between the oscillating tip height $z_t(t)$ and the force $F_Q(t)$, as the latter depends on the charge occupation probabilities $p_A(t)$ and $p_B(t)$.

The link of the effective switching rate $<k_{ET}>$ to the phase shift can be demonstrated by looking at the Fourier component of $F_Q(t)$ that corresponds to the base cantilever frequency f_r. Suppose we take $F_Q(t) \propto \cos(2\pi f_r t + \varphi)$, substitute this into the expressions for the frequency shift and the dissipated energy, equations (2) and (3) of this SOM, respectively. We interpret the resulting Δf and E_{diss} as the peak values associated with the charge switching signal and insert them into the definition of $<k_{ET}>$, Eq. (1) from the main paper. We obtain

$$< k_{ET} > = \pi f_r \operatorname{cotg} \varphi \approx {}^{1}\!/_{2\tau}; \qquad (21)$$

where φ is the phase shift with respect to the tip position $z_t(t)$ and therefore also with respect to $\Delta E(t)$, while τ is the mean time it takes for the charge to switch into the new location after the tip passes the position where $\Delta E = 0$. The condition $\Delta E = 0$ defines the situation in which the bottom of both parabolic potentials for the two redox sites are aligned on the same level, as depicted in Fig. 3b of the main text. The approximate expression for $<k_{ET}>$ that involves the mean delay τ is valid as long as the charge position switches in (almost) every cycle of the tip oscillation and the switching happens relatively soon after $\Delta E = 0$ as compared to the whole oscillation period. Such conditions correspond to a small phase shift φ. That the assumption of small φ was indeed fulfilled could be checked *a posteriori* by observing that $<k_{ET}>$ evaluated from our measured data was several times larger than f_r. Also note that our $<k_{ET}>$ corresponds to one half of Γ_Σ from [Roy-Gobeil, A., Miyahara, Y. & Grutter, P. *Nano Lett.* **15,** 2324–2328 (2015)], eq. (6). Finally, let us note that $<k_{ET}>$ would have a straightforward interpretation in terms of the immediate transfer rates k_{AB} and k_{BA} only if the sum $k_{AB} + k_{BA}$ were constant (or at least only slowly varying with tip oscillations), in which case $<k_{ET}> \approx (k_{AB} + k_{BA}) / 2$. This however definitely does not hold in our model of the transfer mechanism, as we discuss further below.

The rate $<k_{ET}>$ is a characteristic time scale of the charge switching, one which can be extracted from the experimental data in a straightforward manner. However, the interpretation of $<k_{ET}>$ in

terms of the physical parameters of our theoretical model is far from being straightforward. This in particular holds about the connection between $<k_{ET}>$ and the actual time- and configuration-dependent switching rates $k_{AB}(q, t)$ and $k_{BA}(q, t)$, as given by Eq. (2) in the main paper or Supplementary Eqs. (12). On the one hand, the rates k_{AB} and k_{BA} go from 0 to ~20 MHz in our model, as one can see in Fig. 3c, while the effective rate $<k_{ET}>$ calculated from Eq. (1) gives values in the range of 6–8 MHz for different tip-sample distances, so these quantities are at least of the same order of magnitude. On the other hand, the dependence of $<k_{ET}>$ on $k_{AB}(q, t)$ and $k_{BA}(q, t)$ is significantly nonlinear.

The crucial observation for establishing a relation between these rate quantities is the inverse proportionality between $<k_{ET}>$ and the mean delay τ of the charge switching. So as not to complicate the discussion any further, we will assume here that, in accord with Supplementary Eqs. (12) in Supplementary Note 2, the rate k_{BA} is the same function of ΔE_{AB} as the opposite rate k_{AB} is of minus ΔE_{AB}. Furthermore, most of the time, switching in one direction is much more probable than in the opposite direction. So let us only care about one of the possible directions of the switching when estimating τ, say from B to A. The DOF described by the coordinate q is not directly related to the force $F_Q(t)$. Only the location of the charge either on site A or site B decides about the force felt by the tip. We may therefore consider rates integrated over q, such as $k_{AB}(t) = \int dq\, \rho_B(q)\, k_{AB}(q, t)$, for the present discussion. Note that this is also the meaning of the rates shown in the middle panel of Fig. 3c in the main paper.

Now, there are two interwoven trends how $k_{AB}(t)$ affects τ and thus $<k_{ET}>$. First, $<k_{ET}>$ in general grows with growing $k_{AB}(t)$: when k_{AB} is larger, the transfer of the charge to the other redox center occurs sooner, so τ becomes shorter and $<k_{ET}>$ larger. If $k_{AB}(t)$ were roughly constant during the relevant phases of tip oscillation in which switching from B to A occurs, $<k_{ET}> \approx k_{AB}(t)$ would hold. Second, in our model, $k_{AB}(t)$ is in fact not constant but rather changes, often quite abruptly, and peaks for some $\Delta E = \Delta E_{peak}$, somewhere in the range $\Lambda < |\Delta E_{peak}| < (\Lambda + E_{\Lambda})$. If the peak of the transfer rate is sharp (typically when $k_B T << \Lambda$), we may assume that the charge switches exactly when $\Delta E = \Delta E_{peak}$ and then $<k_{ET}> \approx 2\pi f_r\, (\partial E/\partial z)\, A/\Delta E_{peak}$, where A is the oscillation amplitude. If, however, the rates k_{AB} (k_{BA}) exhibit rather broad peaks as functions of ΔE, as it is also the case in our study, the two above described trends participate in determining the value of the effective rate $<k_{ET}>$ in a rather tangled way.

To conclude, in our model the effective rate $<k_{ET}>$ expresses, in a certain way, the phase shift between tip oscillation and the electron transfer switching. Although the phase shift is associated with the $k_{AB}(t)$ and $k_{BA}(t)$ rates, $<k_{ET}>$ depends not only on the magnitude of $k_{AB}(t)$ and $k_{BA}(t)$, but also on how they change in time during tip oscillation. The sooner $k_{AB}(t)$ or $k_{BA}(t)$ grows after passing the equilibrium position, the larger $<k_{ET}>$ becomes. Moreover, the effective rate $<k_{ET}>$ depends non-linearly on how large $k_{AB}(t)$ and $k_{BA}(t)$ rates are. In other words, $<k_{ET}>$ gives only an approximate estimate of the switching rate in our model and it cannot provide direct information about time-dependent character of the $k_{AB}(t)$ and $k_{BA}(t)$ rates employed in the model.

Supplementary Note 9: Amplitude dependence of $\Delta f(z)$ and $E_{diss}(z)$ in experiment and theory

To see how the character of singularity line depends on the oscillation amplitude, we performed series of constant-height measurements, where the oscillation amplitude was gradually increased. Supplementary Figures 13 and 14 show variation of the frequency shift and the dissipation signal in the amplitude range of 40–90 pm and 20–80 pm, respectively. In these amplitude ranges, we observe a modest dependence of the signal on the amplitude. Namely, the width of the frequency shift and the dissipation peaks increases slightly with the amplitude. In the case of the dissipation signal, an enhancement of the maximum value of dissipation signal with the amplitude is more pronounced than in the case of the frequency shift which remains almost constant. Note, tip height is not exactly maintained during variation of the oscillation amplitude due to a feedback response. Thus, the images shown on Supplementary Fig. 13 and 14 are not taken with exactly the same tip height.

In the next, let us compare the numerical results obtained with the full and Marcus model. Despite the fact that the theoretical plots show the tip-height profile, while the experimental profiles are displayed as lateral profiles acquired at the constant tip-height, we can directly compare the shape of the peaks (mainly width and the maximal value of the peaks) with respect to the oscillation amplitude.

In both models, we observe a gradual broadening of the peaks in both channels and increase of the dissipation peaks as the oscillation amplitude increases, in good agreement with the experimental evidence. However, the full model with both the Marcus and explicit DOF shows better match with the experimental evidence. Namely, the experimental data presented in Supplementary Fig. 13 and 14 are taken in far tip-sample distance regime, as the characteristic circular features reflecting the ferrocene units are not visible. The evolution of simulated frequency shift peaks with the amplitude in far tip-sample distances (two peaks on the right in Supplementary Fig. 11a) matches perfectly the corresponding evolution observed in experiment. First, the maximum of the frequency shift increases from A = 20 pm to 40 pm, while for the amplitude A = 80 pm it drops again and the peak becomes broader. This behavior cannot be reproduced in the Marcus model, where the frequency shift monotonically decreases with larger amplitude, see Fig. 5c. We also think that the evolution of the dissipation signal with the oscillation amplitude is better captured by the full model. Thus, the amplitude dependence provides another indication, that the full model with the Marcus and explicit DOF describes better the electron transfer mechanism, which takes place in our system.

Supplementary Figures

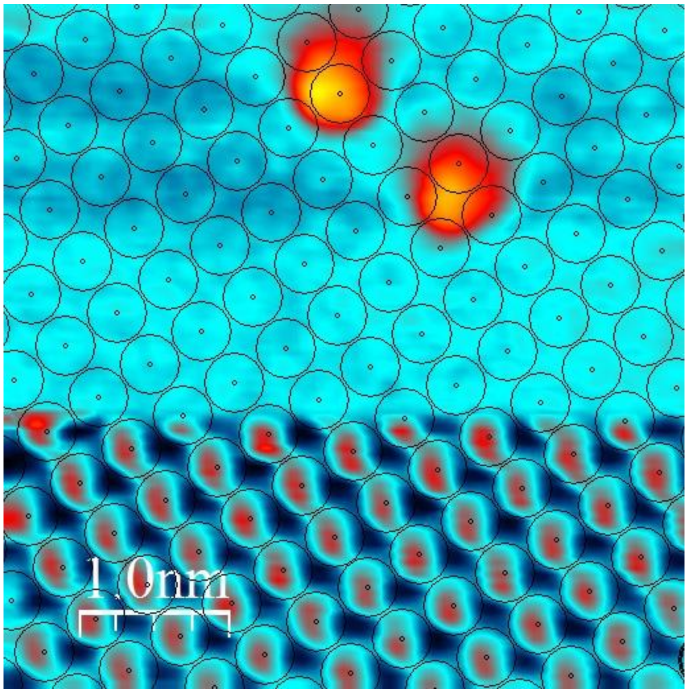

Supplementary Figure 1. Register with substrate. Constant height frequency shift image of bisFc molecule with atomic resolution of NaCl substrate. Centers of circles denote Cl atoms. Image was taken in two different tip - sample heights. First, the resolution on substrate was taken, then the tip was retracted by 0.6 nm and the bisFc molecule was imaged. Scanning area is 4.5 × 4.5 nm^2, oscillation amplitude A = 40 pm, bias voltage U = 0 V.

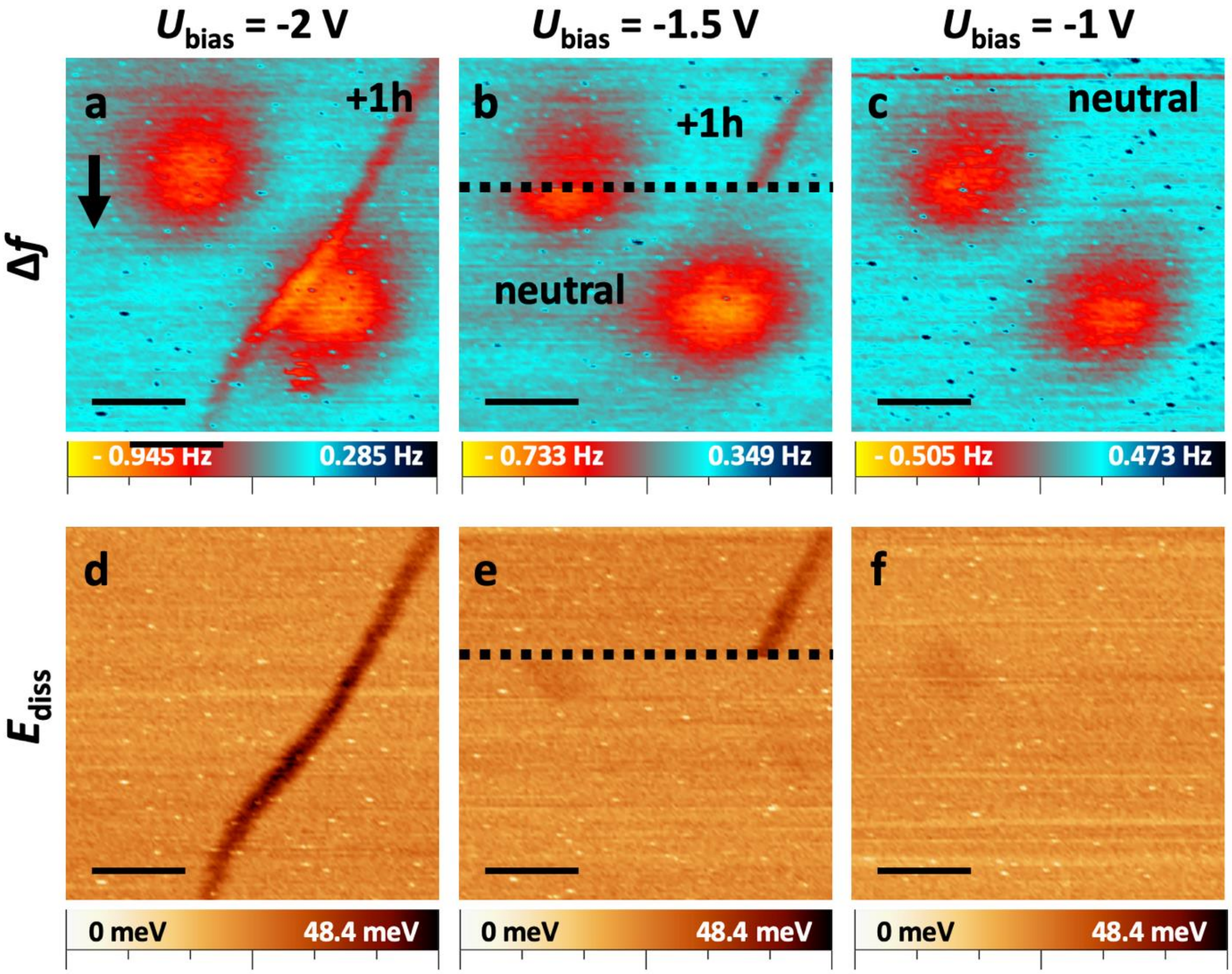

Supplementary Figure 2. An example of discharging process of bisFc molecule. **(a)-(c)** and **(d)-(f)** A simultaneously taken series of the constant height frequency shift and dissipation images taken at same tip-sample distance at three different bias voltages: $U = -2$ V, $U = -1.5$ V and $U = -1$ V (corresponding to once positively charged molecule (a) and neutral molecule (b) and (c)). The scanning area is 2×2 nm^2, scale bar denotes 500 pm, oscillation amplitude $A = 50$ pm for each image, temperature 5 K. Scanning direction in all images is from top to bottom. Dashed line in (b) and (e) denotes the line where discharging event happened.

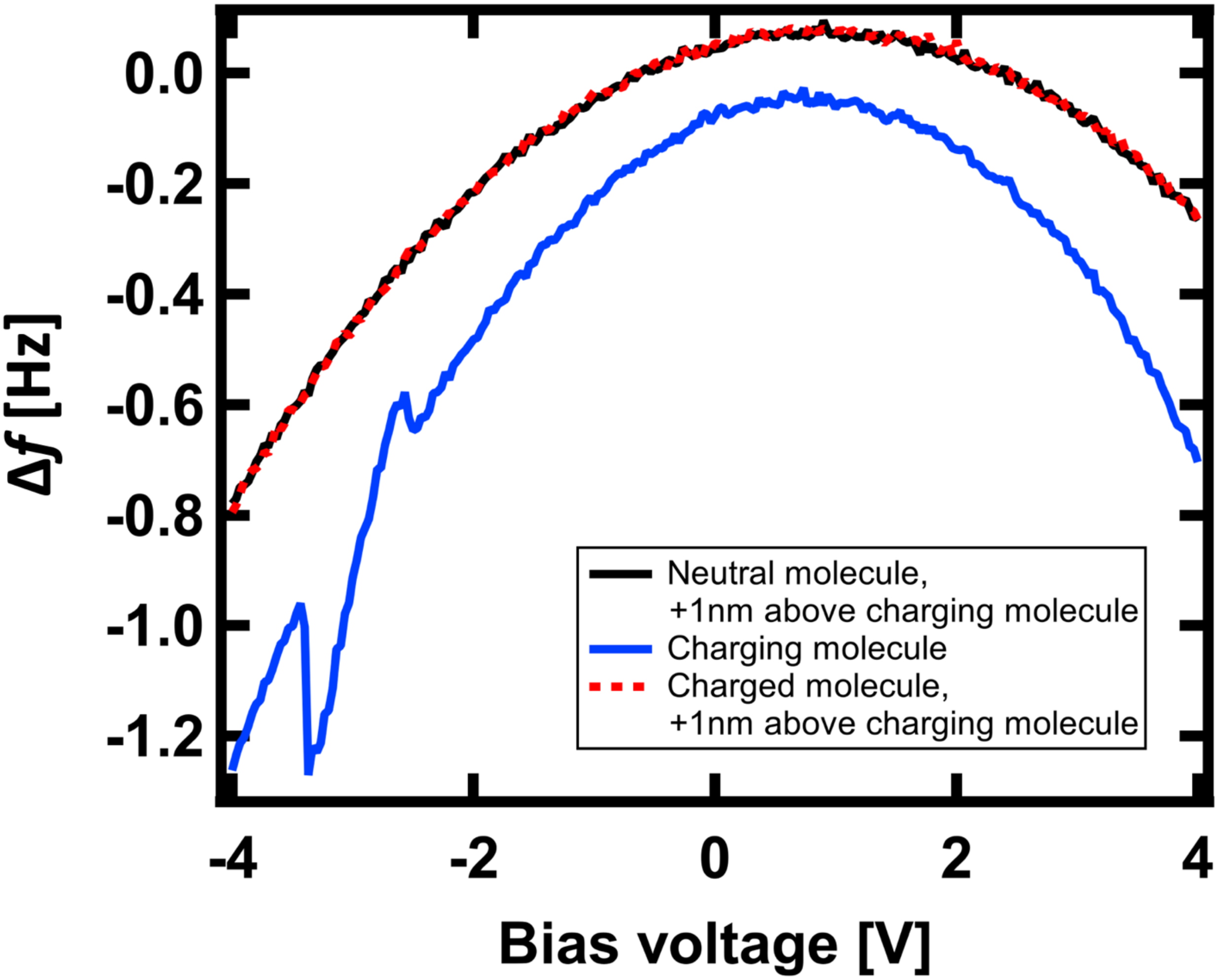

Supplementary Figure 3. Kelvin parabolas above neutral and once positively charged molecule. Series of $\Delta f(U)$ parabolas taken in 2 different tip-sample heights. Black solid line represents a parabola taken above bisFc molecule in the neutral state at relative height z =+1 nm. Blue solid line represents a charging parabola taken at relative height z = 0 nm (1 nm closer to the bisFc molecule); bisFc becomes twice positively charged during its acquisition. Red dotted parabola is taken at relative height z =+1nm above twice positively charged bisFc molecule. Oscillation amplitude is A = 50 pm.

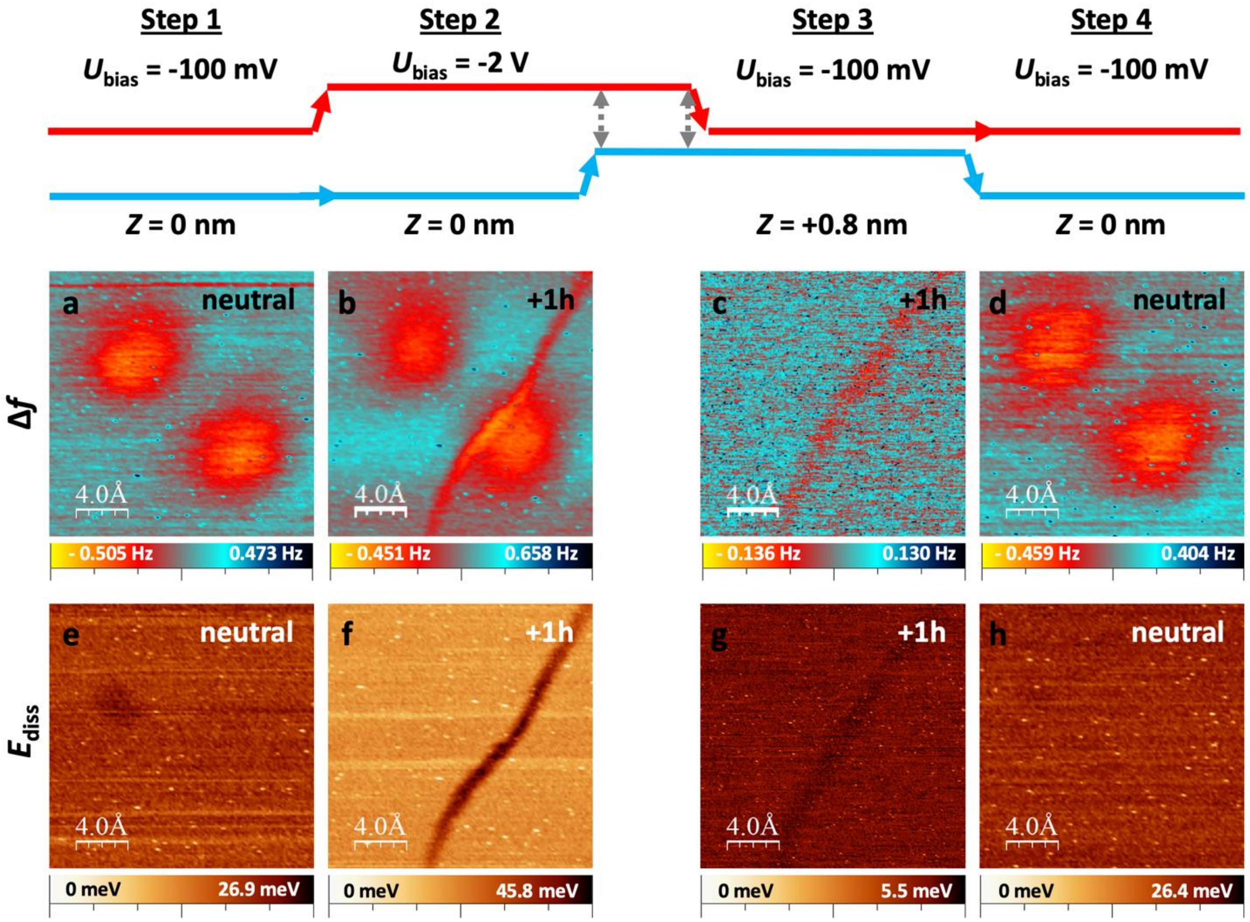

Supplementary Figure 4. Recognition of charged bisFc molecule in far distance. **(a)-(d)** and **(e)-(h)** A simultaneously taken series of the constant height frequency shift and dissipation images taken at two different tip-sample distances and at two different bias voltages: $U = -100$ mV (panels (a), (e), (c), (g), (d), (h), bias corresponds to a neutral molecule) and $U = -2$ V (corresponds to a once positively charged molecule, panels (b), (f)). The scanning area is 2×2 nm^2, oscillation amplitude $A = 50$ pm for each image, temperature 5 K. Scanning direction in all images is from top to bottom. Step 1 **(a,e)** presents a neutral molecule at a relative tip-sample distance $z = 0$ nm. Step 2 **(b,f)** shows a once positively charged molecule (bias voltage $U = -2$ V) at same relative tip sample distance $z = 0$ nm. Between step 2 and step 3 the tip is retracted by $z = +0.8$ nm while keeping bias voltage $U = -2$ V. Step 3 **(c,g)** represents charged molecule at "far" distance, relative tip sample distance $z = +0.8$ nm, scanned at bias voltage $U = -100$ mV that corresponds to bias were the molecule is normally found in the neutral state, but in this particular case discharging is prevented by the tip-sample distance. Step 4 **(d,h)** shows images taken at close distance, relative tip sample distance $z = 0$ nm, at bias voltage $U = -100$ mV (bisFc molecule is in neutral charge state again).

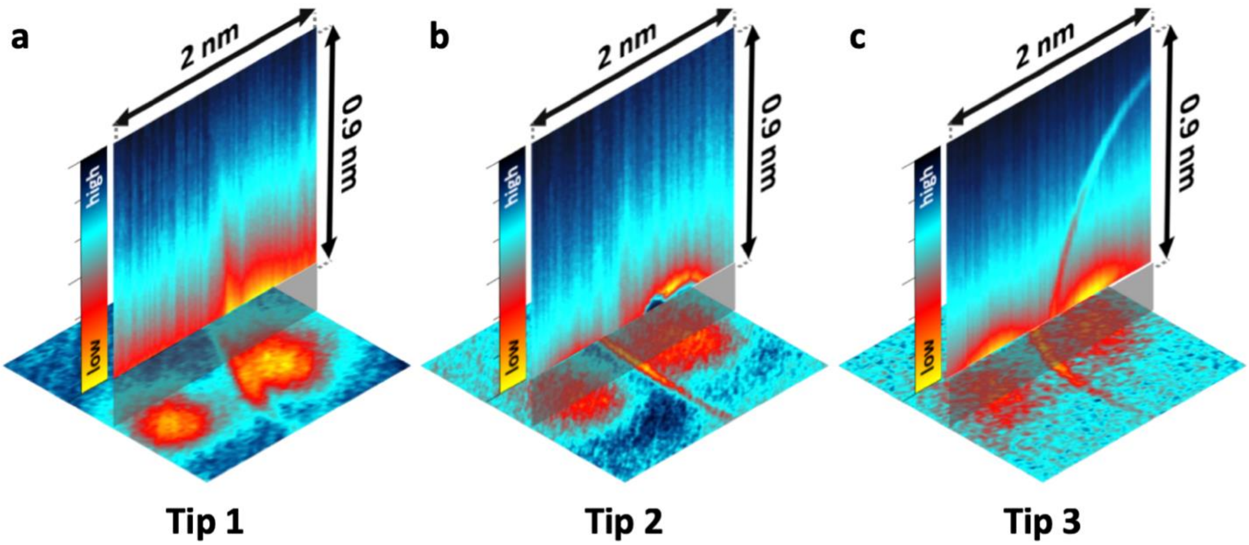

Supplementrary Figure 5. Dependence of Δf line shape on tip apex. **(a)-(c)** Frequency shift $\Delta f(z)$ *x*-*z* images of excitation line (once positively charged molecule) with different tip apexes above the same molecule with the same bias voltage $U = -2.5$ V. **(a)** Tip 1—metallic tip, **(b)** tip 2—after interaction with NaCl substrate, **(c)** tip 3—after another interaction with NaCl substrate. The vertical cuts are 2×0.9 nm^2 with the gap of 0.1 nm between base images and perpendicular images of singularity line. The perpendicular image is taken with bias voltage $U = -2.5$ V. Base images of bisFc are constant height frequency shift images, taken with bias voltage $U = -2.5$ V (once positively charged molecule). Scanning area of base images is 2×2 nm^2.

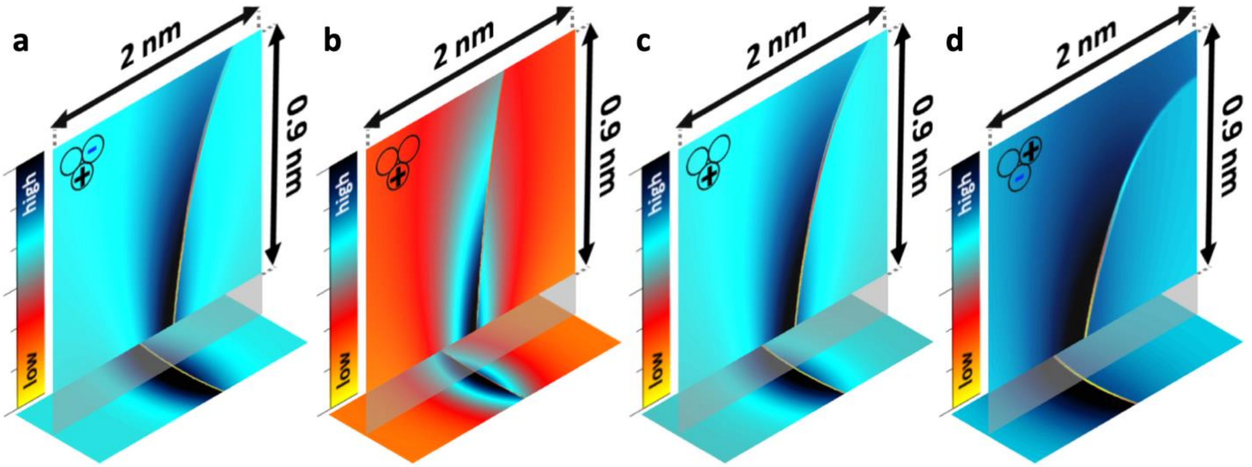

Supplementary Figure 6. Simulated $\Delta f(z)$ lines with different tip apex charges. **(a)-(d)** Simulated frequency shift *x*-*z* images of excitation line (once positively charged molecule) with different charge in tip apex. The vertical cut is 2×0.8 nm^2, the area of base images is 2×0.8 nm^2. All images were calculated for temperature $T = 1.2$ K. **(a)** The tip is composed from two point charges, an apex charge of +0.50 and a side charge −0.25. The side charge is shifted by 1 Å up and 1 Å to the right. The total charge is +0.25. **(b)** Only apex charge +0.25. **(c)** Only apex charge +0.50. **(d)** The same geometry as in (a) but values of apex charge and side charge are swapped and total charge is also +0.25.

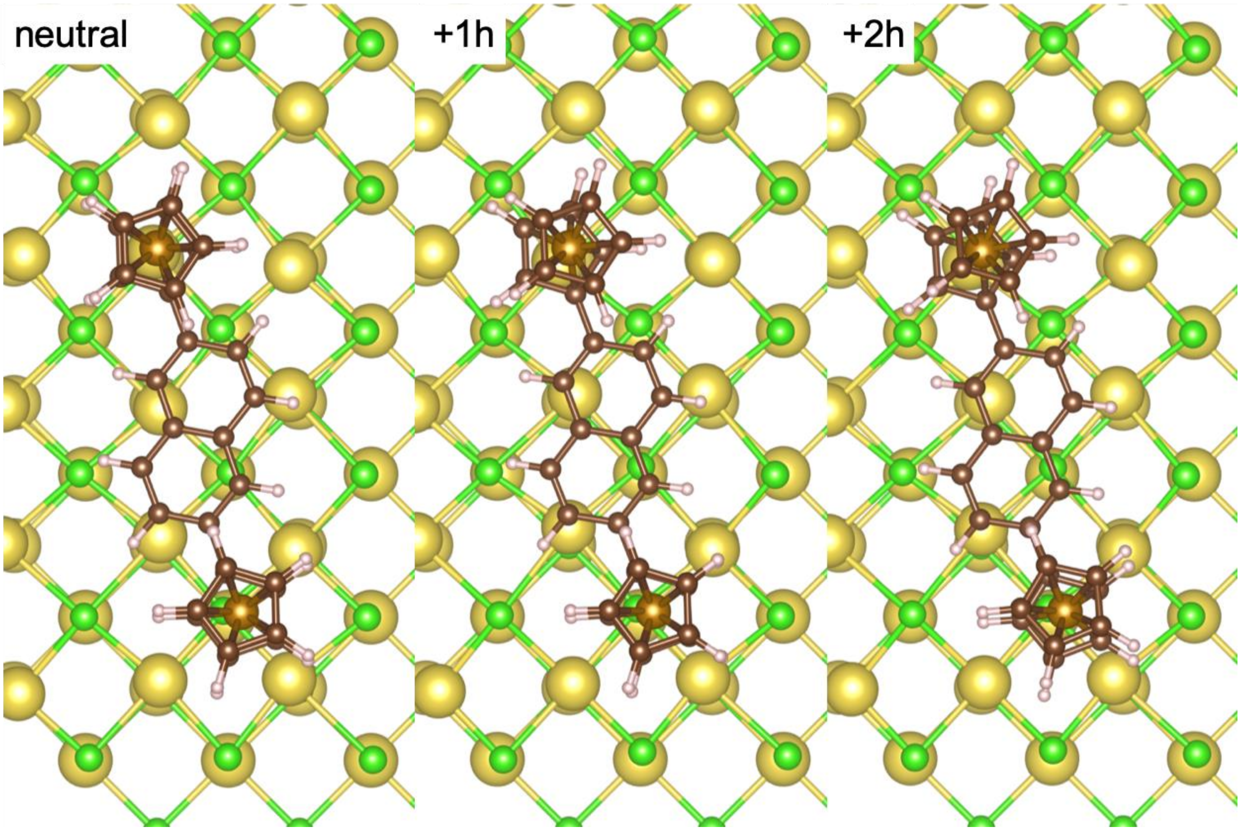

Supplementary Figure 7. Top view of the optimized DFT structure. The optimized structures of neutral, +1h and +2h molecular structures on the NaCl surface is shown. The charged Fc unit changes its position with respect to the surface, adopting a slightly inclined position with respect to it.

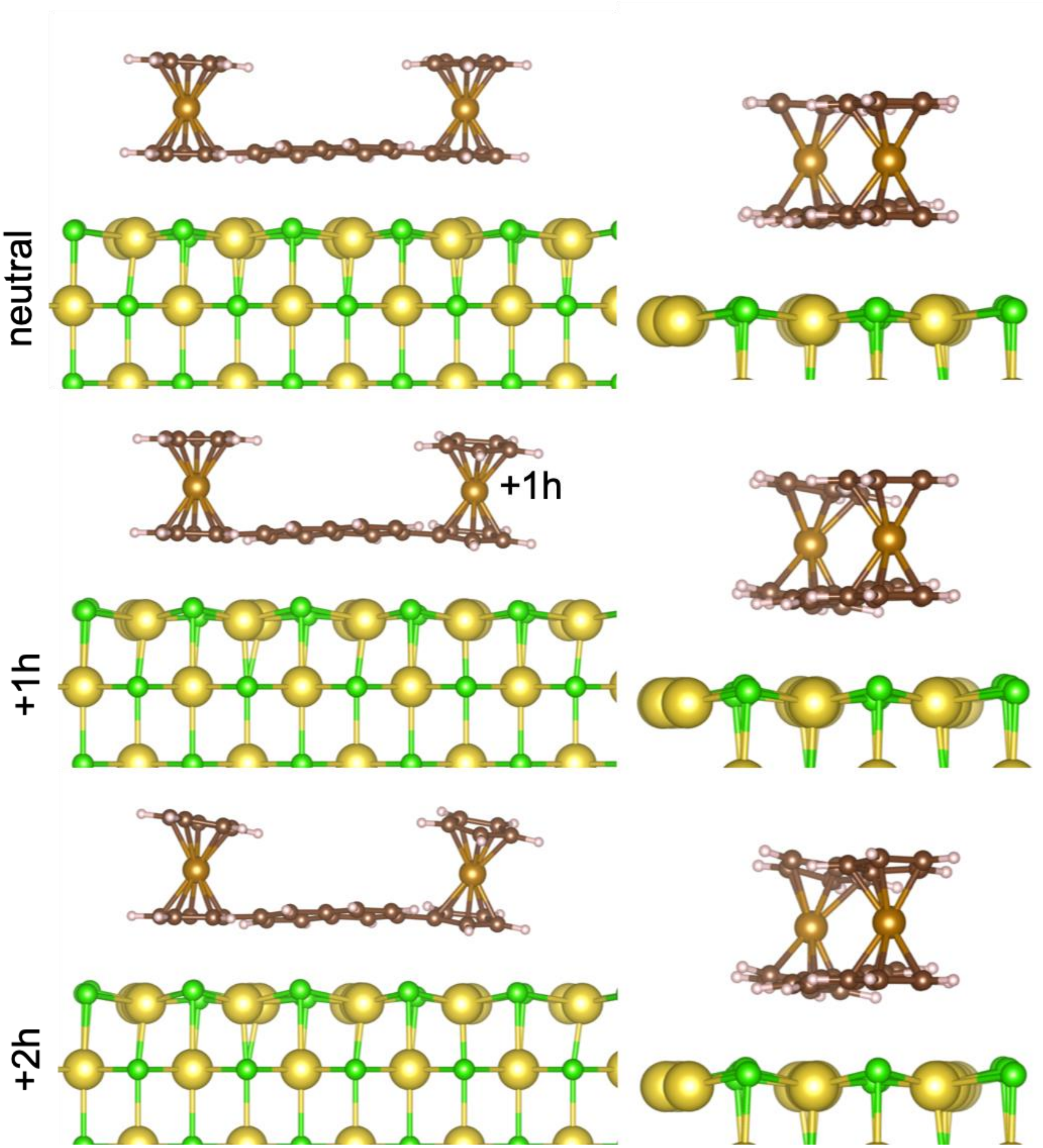

Supplementary Figure 8. Optimized DFT structure; side views. The optimized structures of neutral, +1h and +2h molecular structures on the NaCl surface is shown. The charged Fc unit changes its position, adopting a slightly inclined position with respect to the surface.

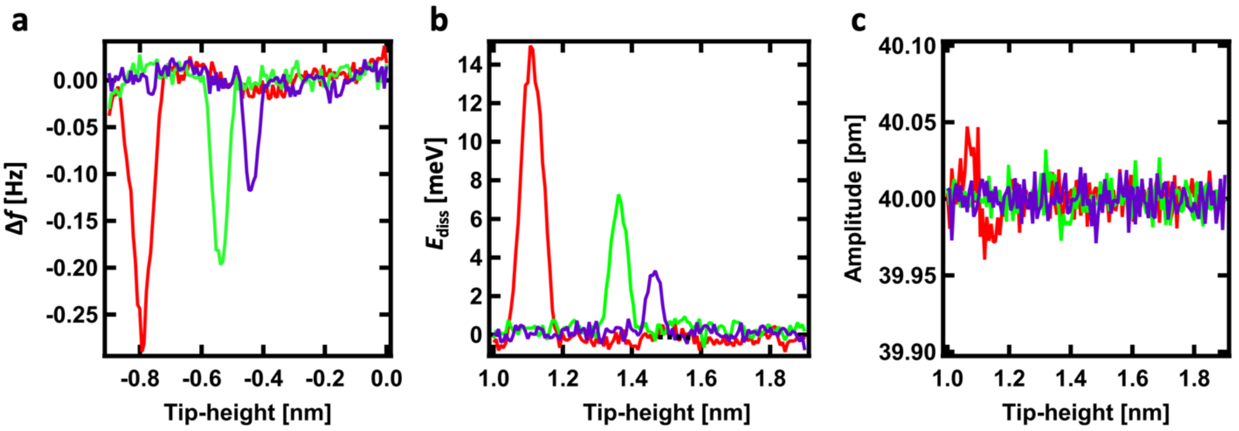

Supplementary Figure 9. Dependence of Δf, E_{diss} and amplitude during tip-height spectroscopy. **(a)** $\Delta f(z)$ spectra taken at three different positions, see the red, green, and purple lines marked in Fig. 2g and Fig. 2h in the main text. Background has been subtracted from all curves. **(b)** Dissipation signal $E_{\mathrm{diss}}(z)$ spectroscopies corresponding to $\Delta f(z)$ spectroscopies. All curves are normalized to background dissipation. **(c)** Value of amplitude corresponding to $\Delta f(z)$ spectroscopies. Oscillation amplitude during acquisition A = 40 pm. The range of amplitudes is within 0.1% of the set point amplitude A = 40 pm.

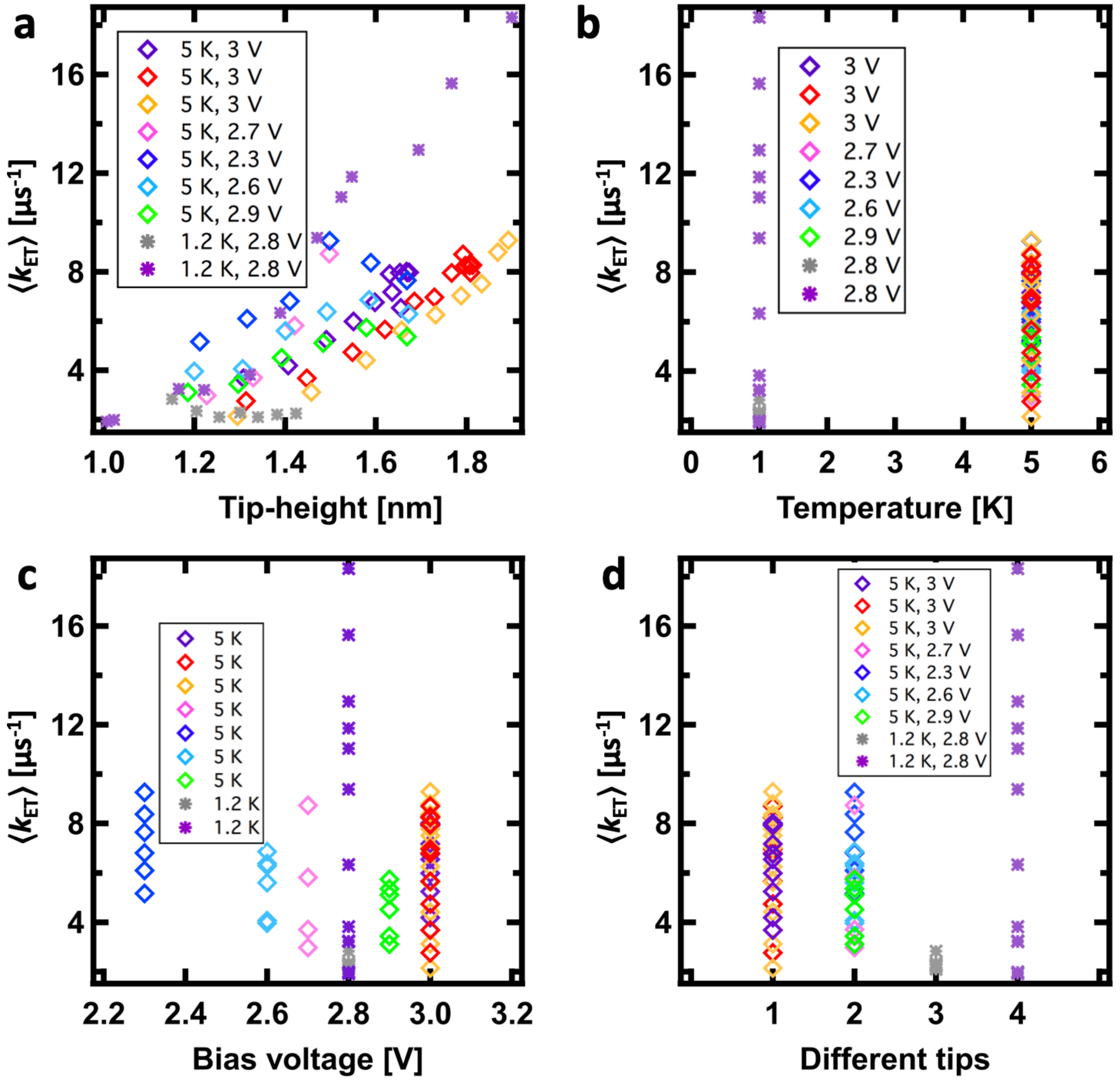

Supplementary Figure 10. Effective transfer rates plotted against tip-sample distance, applied bias voltage, temperature and different tips. **(a)** Identical data of the effective transfer rate $<k_{ET}>$ as shown in Fig. 2k, but replotted against different bias voltages **(b)**, temperatures **(c)** and tips **(d)** obtained during different experimental sessions.

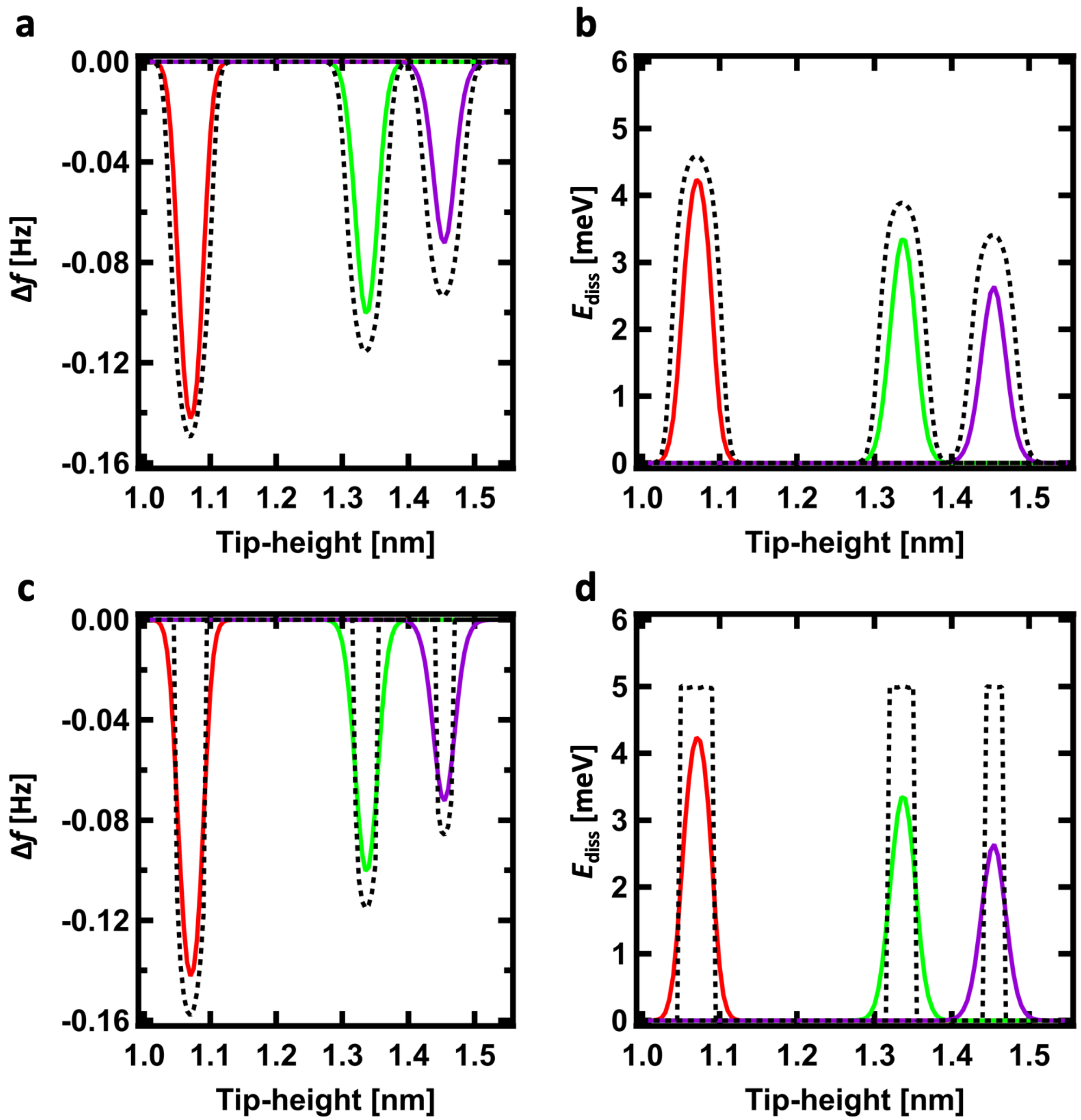

Supplementary Figure 11. Comparison of $\Delta f(z)$ and $E_{\text{diss}}(z)$ line profiles for different numerical models. Frequency shift $\Delta f(z)$ **(a,c)** and dissipated energy $E_{\text{diss}}(z)$ **(b,d)** predicted by the full version of our numerical model (color solid lines) are compared to the results of two simplified models (black dashed lines): The model which disregards the "explicit" degrees of freedom and retains only the Marcus ones **(a,b)**, and the model with the threshold energy for electron transfer **(c,d)**. Parameters of the full model were $E_\Lambda = \Lambda = 4\times10^{-22}$ J ≈ 2.5 meV, $K_\Lambda = 2\times10^{6}$ s^{-1}, $k_0 = 3.46\times10^{7}$ s^{-1}; parameters of the all-Marcus model $\Lambda = 6\times10^{-22}$ J ≈ 3.75 meV, $k_0 = 2.45\times10^{7}$ s^{-1}; for the model with a threshold energy for electron transfer $\delta E = 4\times10^{-22}$ J ≈ 2.5 meV. All simulations were done at T = 5 K. In all three cases, the same model was applied to represent the tip and the same parameters of the oscillator were used (f_r = 1 MHz, k = 1 MN m^{-1}, Q_t = +0.25 e).

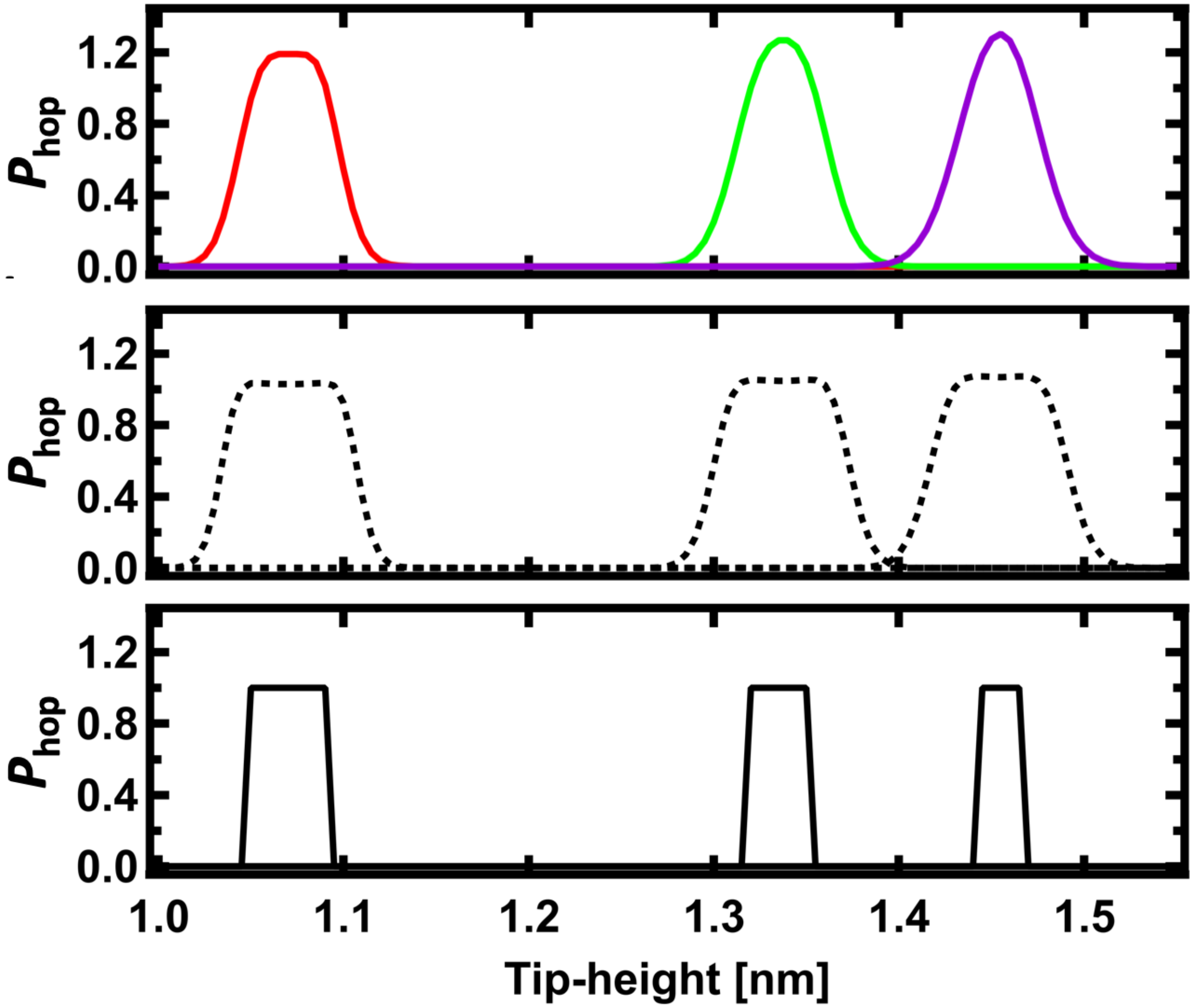

Supplementary Figure 12. Comparison of average number of electron transfer events for different numerical models. Average number of electron transfer events (in one direction, say A→B) per one period of tip oscillation ("hopping probability") as a function of the mean position around which the AFM tip oscillates. **(a)** the full model with the Marcus and explicit DOF, **(b)** all-Marcus model without the explicit DOF and **(c)** threshold-energy model. While the number of electron transfer events peaks in all models whenever the energy difference between the two redox sites oscillates around zero, peak shapes distinctly differ between the three models. Model sensitivity of peak shapes in this quantity translates into model sensitivity of peak shapes in the measurable signal, i.e. in the frequency shift $\Delta f(z)$ and, mainly, the dissipated energy $E_{\mathrm{diss}}(z)$ as illustrated by the previous Supplementary Figure 11.

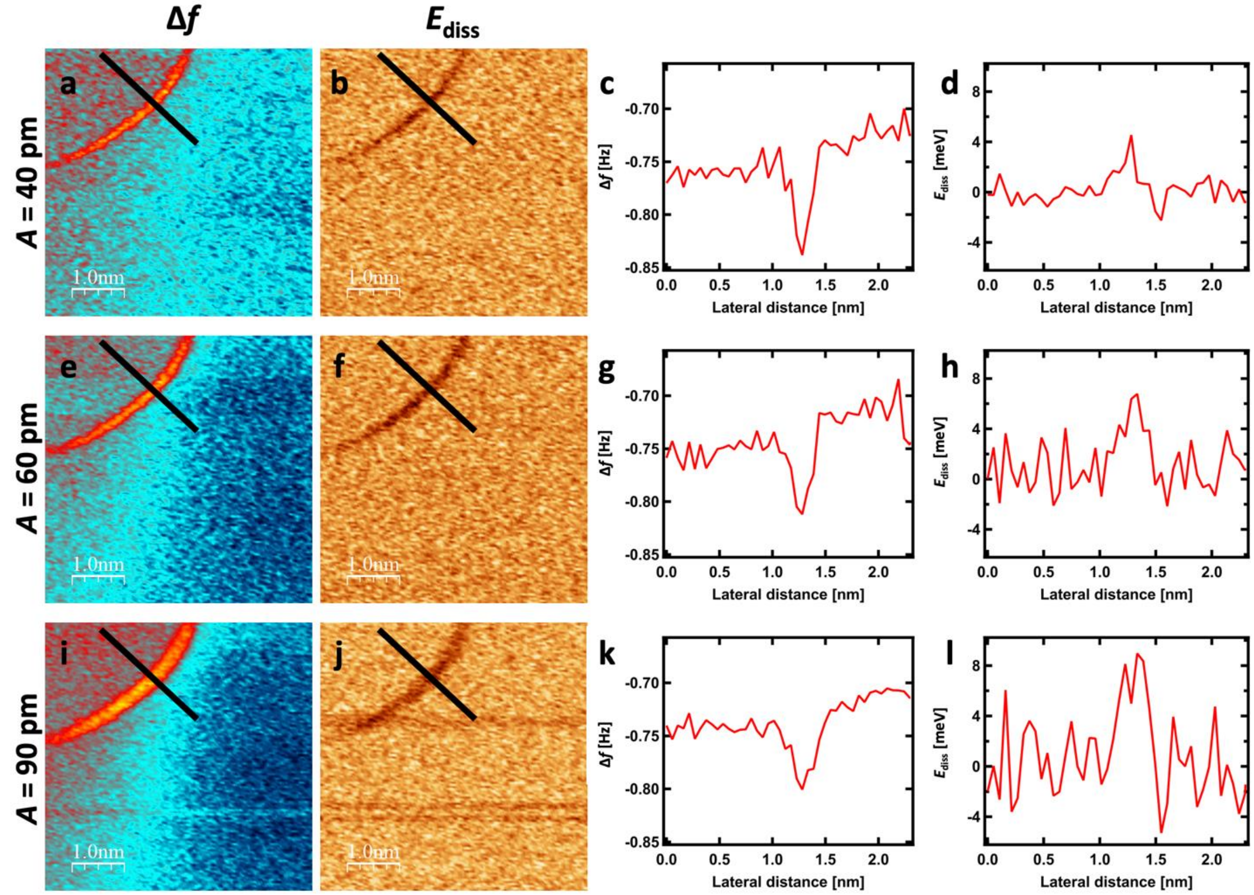

Supplementary Figure 13. Amplitude dependence. **(a,b)**, **(e,f)** and **(i,j)** Series of constant height frequency shift and dissipation images taken at constant tip-sample distance with three different amplitudes of oscillation: A = 40 pm, A = 60 pm and A = 90 pm at bias voltage: $U = -3.8$ V (corresponding to once positively charged molecule). The scanning area is 5×5 nm^2. **(c,d)** A = 40 pm, **(g,h)** A = 60 pm and **(k,l)** A = 90 pm line profiles of frequency shift and dissipation signal corresponding to the constant height images. The profiles are denoted by black lines in images.

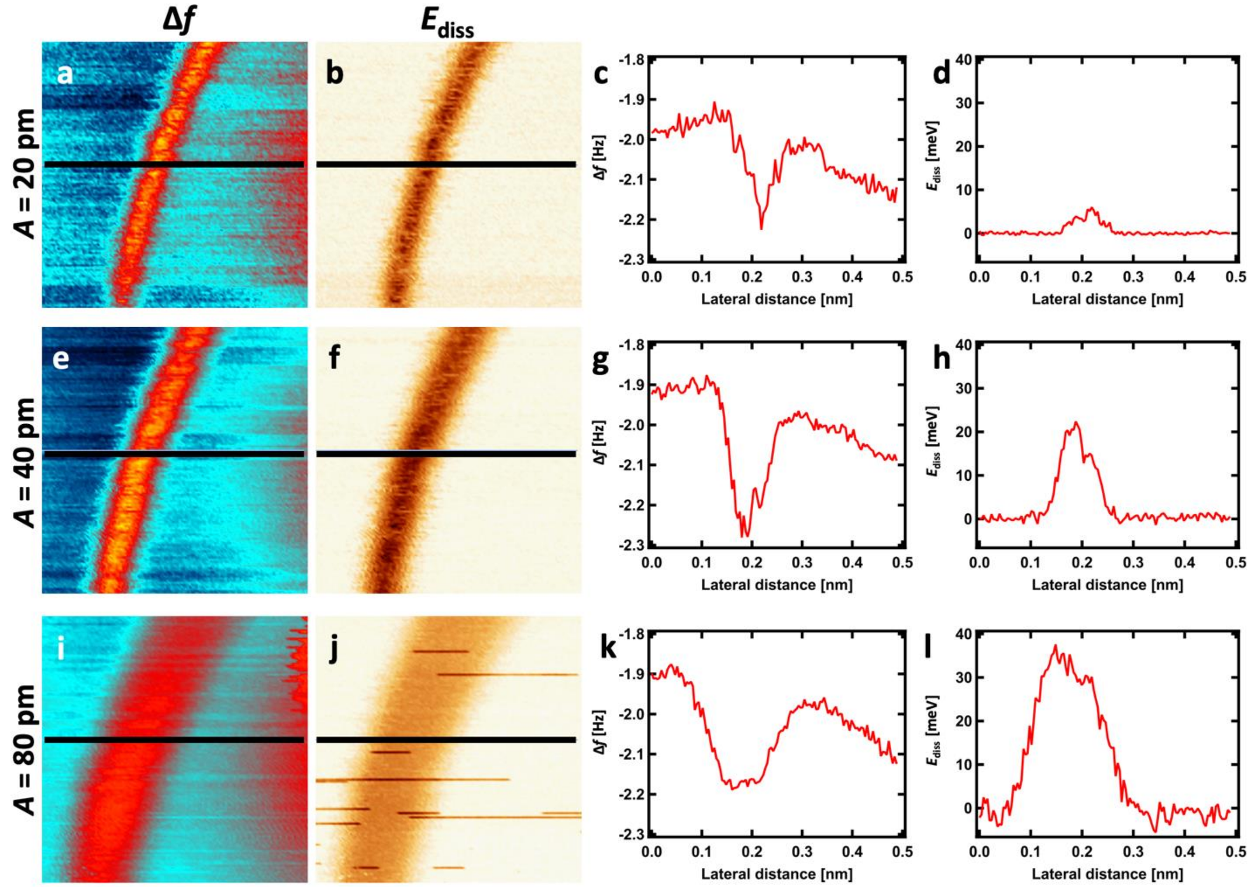

Supplementary Figure 14. Amplitude dependence. **(a,b)**, **(e,f)** and **(i,j)** series of constant height frequency shift and dissipation images taken at approximately constant tip-sample distance with different amplitudes of oscillation: A = 20 pm, A = 40 pm and A = 80 pm at bias voltage: $U = -2.6$ V (corresponding to once positively charged molecule). The scanning area is 0.5×0.5 nm^2. **(c, d)** A = 20 pm, **(g, h)** A = 40 pm and **(k, l)** A = 80 pm line profiles of frequency shift and dissipation signal corresponding to the constant height images. The profiles are denoted by black lines in the images.

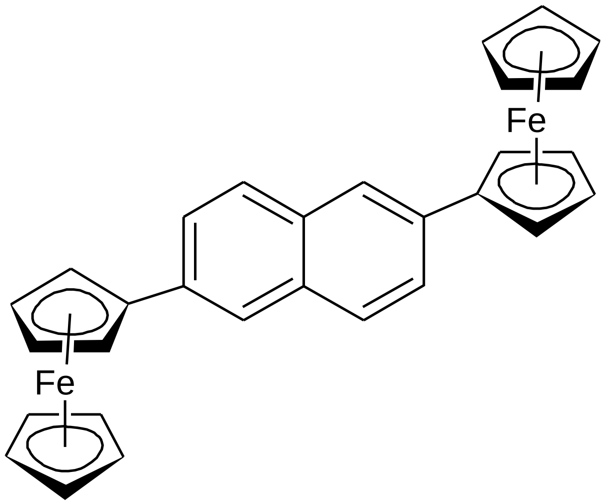

Supplementary Figure 15. 2,6-Diferrocenylnaphthalene (mentioned without the synthesis in a patent: JP 2005-322787)

A Schlenk flask equipped with a reflux condenser was charged with 2,6-dibromonaphthalene (100 mg, 0.350 mmol), ferroceneboronic acid (322 mg, 1.40 mmol, 4.0 equiv.), silver oxide (178 mg, 0.770 mmol 2.2 equiv.), tetrakis(triphenylphosphine)-palladium(0) (40 mg, 0.035 mmol, 0.1 equiv.) and silver fluoride (213 mg, 1.40 mmol, 4 equiv.), evacuated and re-filled with argon three times. Degassed distilled tetrahydrofuran (20 mL) was then added, the suspension was refluxed for 3 h and then heated to 65 °C overnight. After cooling back to room temperature the mixture was filtered through a pad of silica gel. Evaporation of the solvent in *vacuo* afforded dark orange crude product, which was subjected to flash column chromatography on silica gel (hexane–dichloromethane 100:0 to 50:50) to give 109 mg (55 %) of orange powder. Final purification was achieved by vacuum sublimation (220 °C, 10^{-3} mbar).
^{1}H NMR (400 MHz, $CDCl_3$): 4.07 (5 H, s), 4.33–4.42 (2 H, m), 4.74–4.79 (2H, m), 7.63 (1 H, dd, *J* = 8.5, 1.7 Hz), 7.73 (1 H, d, *J* = 8.5 Hz), 7.82 (1 H, d, *J* = 2.0 Hz).
^{13}C NMR (100 MHz, $CDCl_3$): 66.55, 69.11, 68.59, 85.50, 123.48, 125.64, 127.38, 132.31, 136.16.
ESI MS: 496 (M^+), 535 ($[M+K]^+$).
HR ESI MS: calculated for $C_{30}H_{24}Fe_2$ 496.05714; found 496.05642.
Combustion analysis: calculated for $C_{30}H_{24}Fe_2$ 72.62% C, 4.88% H; found 72.46% C, 4.88% H.

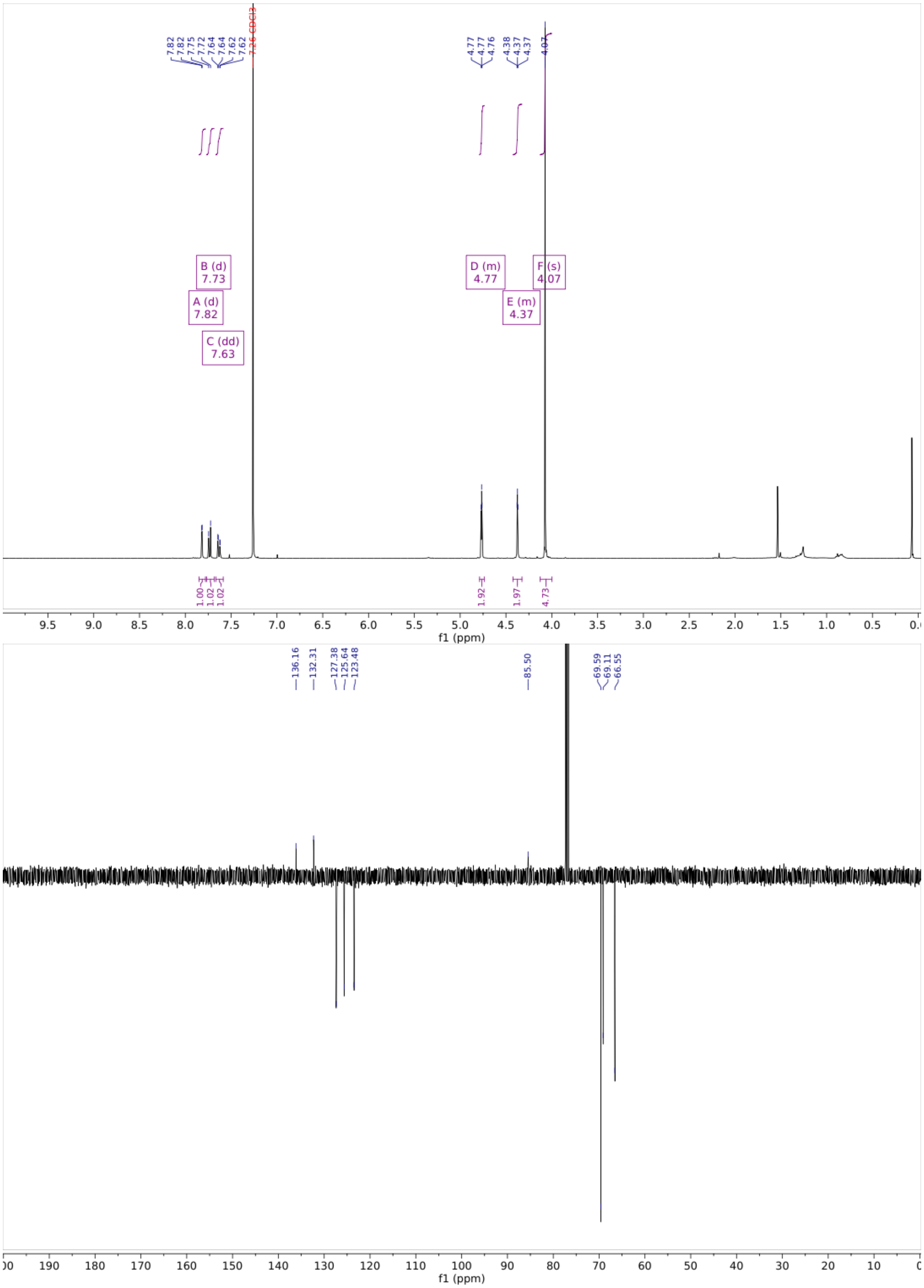

Supplementary Figure 16. ^{1}H and ^{13}C NMR spectra of 2,6-diferrocenylnaphthalene (bisFc).

Supplementary Movie 1. Illustriation of the charge transfer dynamics in our model.
Animation of dynamics in our ET model during one cycle of the oscillating AFM tip corresponding to simulations presented in Fig. 4c,d in the main text. Panels on the left: On the top, probability that the charge occupies the respective redox site A (blue curve) or B (red curve). In the middle, the momentary transfer rate from A to B (red) and back (blue). At the bottom, difference between the two sites in terms of total energy cost of being occupied by the charge. This difference is directly determined by the oscillating tip. Panel on the right: Evolution of parabolic potential-energy surfaces (dotted lines) and the probability densities (solid lines) that describe the charge location (integral under the respective curves) and relaxation of the molecule in terms of the “explicit’’ degrees of freedom (q) (shape of the “wave packets”). Blue curves relate to site A, red curves to site B. The temperature is 5 K for the case shown here. Model parameters are $E_\Lambda = \Lambda = 4\times10^{-22}$ J ≈ 2.5 meV, $K_\Lambda = 2\times10^{6}$ s^{-1}, $k_0 = 3.46\times10^{7}$ s^{-1}, oscillation amplitude A = 40 pm.
