# Supplementary material for: Quantum dissipation driven by electron transfer within a single molecule investigated with atomic force microscopy
Source: Nat Commun. 2020 Mar 12;11:1337. doi: 10.1038/s41467-020-15054-w (PMC7067884; doi:10.1038/s41467-020-15054-w)
Supplement: Supplementary file 3 — Description of Additional Supplementary Information [file 41467_2020_15054_MOESM3_ESM.pdf]

Description of Additional Supplementary Files

File Name: Supplementary Movie 1.

Description: Illustration of the charge transfer dynamics in our model.

Animation of dynamics in our ET model during one cycle of the oscillating AFM tip corresponding to simulations presented in Fig. 4c, Fig. 4d in the main text. Panels on the left: On the top, probability that the charge occupies the respective redox site A (blue curve) or B (red curve). In the middle, the momentary transfer rate from A to B (red) and back (blue). At the bottom, difference between the two sites in terms of total energy cost of being occupied by the charge. This difference is directly determined by the oscillating tip. Panel on the right: Evolution of parabolic potential-energy surfaces (dotted lines) and the probability densities (solid lines) that describe the charge location (integral under the respective curves) and relaxation of the molecule in terms of the “explicit’’ degrees of freedom (q) (shape of the “wave packets”). Blue curves relate to site A, red curves to site B. The temperature is 5 K for the case shown here. Model parameters are EΛ = Λ = 4×10-22 J ≈ 2.5 meV, KΛ = 2×106 s-1, k0 = 3.46×107 s-1, oscillation amplitude A = 40 pm.
