# Supplementary figures and images for: Quantum dissipation driven by electron transfer within a single molecule investigated with atomic force microscopy
Source: Nat Commun. 2020 Mar 12;11:1337. doi: 10.1038/s41467-020-15054-w (PMC7067884; doi:10.1038/s41467-020-15054-w)

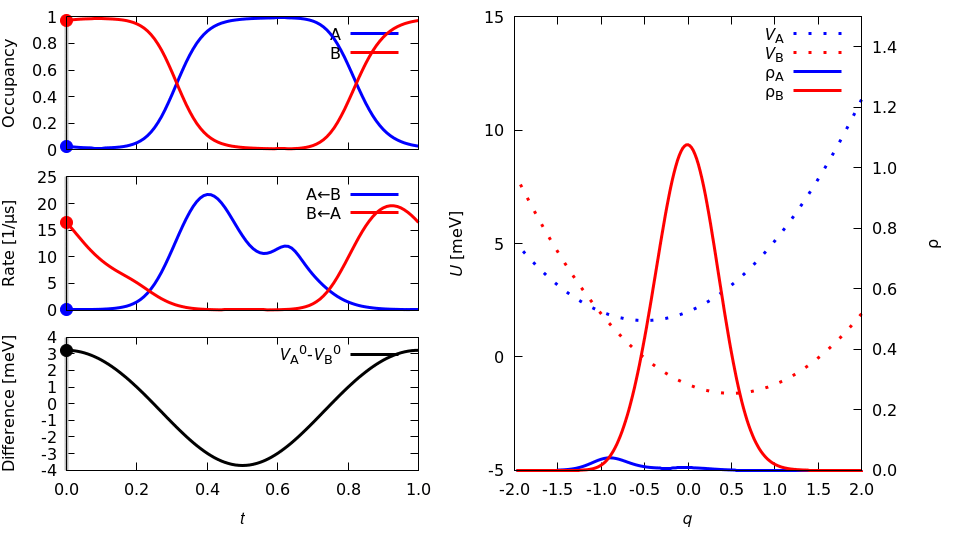

Supplement: Supplementary file 4 — Supplementary Movie 1 [file 41467_2020_15054_MOESM4_ESM.gif]
